# Adsorption of Pb^2+^ ion using coal fly ash/g-C_3_N_4_/Bi_2_O_2_CO_3_ coupled with photocatalytic and forensic applications of spent adsorbent

**DOI:** 10.1007/s11356-026-38106-y

**Published:** 2026-08-22

**Authors:** M. E. H. Ahamed, Kriveshini Pillay

**Affiliations:** 1 Department of Chemical Sciences, University of Johannesburg, Doornfontein, Johannesburg, 2028 South Africa; 2 Department of Chemistry, College of Science, Sudan University of Science and Technology, Khartoum, Sudan

**Keywords:** Pb^2+^, Coal fly ash, Spent adsorbent, Central composite design, Photocatalyst, RhB, Latent Fingerprint

## Abstract

Here, the application of a novel coal fly ash/graphitic carbon nitride/Bismuth oxy carbonate (CFA/g-C_3_N_4_/Bi_2_O_2_CO_3_) composite for Pb(II) removal from aqueous solutions is reported. The composite was characterized by X-ray diffraction (XRD), scanning electron microscopy (SEM), X-ray photoelectron spectroscopy (XPS), and other techniques. The factors affecting the Pb(II) removal performance in batch adsorption experiments, including contact time, adsorbent dosage, and solution pH, were evaluated using the response surface methodology (RSM) approach with central composite design (CCD). The analysis revealed that under optimal conditions for Pb(II) removal (107.6 min, 24.35 mg, pH 6.62, at 25 °C), Pb(II) removal performance reached 100%. Kinetic studies indicated a rapid adsorption process, well-conformed with the pseudo-second order model. Isotherm analysis affirmed the applicability of the Langmuir model, suggesting a monolayer adsorption with a maximum adsorption capacity of 60.29 mg g⁻^1^. The adsorption process was endothermic and spontaneous. The Pb^2+^ ion-loaded spent adsorbent (CFA/g-C_3_N_4_/Bi_2_O_2_CO_3_@Pb^2+^) was then reused to degrade rhodamine B (RhB). The photocatalytic experiments for RhB over CFA/g-C_3_N_4_/Bi_2_O_2_CO_3_@Pb^2^⁺ under optimal conditions demonstrated excellent photocatalytic performance (< 99%) and high reusability (up to 5 cycles). This highlights that the CFA/g-C_3_N_4_/Bi_2_O_2_CO_3_@Pb^2^⁺ is a potential photocatalyst for dye remediation in wastewater treatment applications. Additionally, CFA/g-C_3_N_4_/Bi_2_O_2_CO_3_@Pb^2^⁺ demonstrated to be responsive for latent fingerprint (LFP) detection on a variety of substrates. As a result, it is a useful labeling agent for LFP detection in forensic authentication.

## Introduction

Industrialization and the extensive use of organic dyes have significantly contributed to environmental pollution, particularly through effluents generated by textile, agricultural, mining, electroplating, paint, and battery industries (Joseph & Kim 2026; Ubani-Rex et al. 2017). Among heavy metals, lead (Pb) is of particular concern due to its persistence, non-biodegradability, and severe toxicity, even at low concentrations (Abd El-Fattah et al. 2026; Hill & Gailer 2021). Long-term exposure to Pb can result in neurological, renal, thyroid, and nervous system disorders, highlighting the need for effective monitoring and removal from wastewater (Hill & Gailer 2021; Thamarai et al. 2024; Vukelić et al. 2023).

Adsorption has emerged as a preferred method for heavy metal removal because of its simplicity, low cost, operational efficiency, and potential for adsorbent regeneration (Sukchit et al. 2025). However, the disposal of metal-loaded adsorbents remains a major challenge (Munyengabe et al. 2024). Recent studies have therefore explored the reuse of spent adsorbents in applications such as catalysis, battery production, latent fingerprint (LFP) detection, and construction materials (Mofulatsi et al. 2022). In this context, the present study investigates the reuse of Pb-loaded spent adsorbents as photocatalysts for the degradation of rhodamine B (RhB), aiming to reduce secondary pollution while enhancing resource recovery. Meanwhile, the metal-loaded spent adsorbent can be reused in a latent fingerprint application (Prabakaran & Pillay 2020).

Coal fly ash (CFA), a by-product of thermal power plants, is an attractive low-cost adsorbent due to its abundance, thermal stability, porosity, chemical resistance, and high content of metal oxides (Arofah et al. 2024; Munyengabe et al. 2024; Tenza et al. 2026). Although CFA has demonstrated effectiveness in heavy metal adsorption, its relatively low adsorption capacity necessitates surface modification (Joseph & Kim 2026; Umejuru et al. 2021). Previous studies have shown improved adsorption performance through the incorporation of metal oxides and nanocomposites, including MnO₂ (Mofulatsi et al. 2022), ZnO (Kim et al. 2015), Fe₃O₄, (Arofah et al. 2024) and tungsten oxide–graphene oxide (Umejuru et al. 2021).

Rhodamine B (RhB) is a widely used synthetic dye with toxic, carcinogenic, and mutagenic properties that pose serious environmental and health risks (Ma et al. 2023). Conventional dye removal technologies often suffer from drawbacks such as high cost, sludge generation, and maintenance requirements (Abbas et al. 2021). Photocatalytic oxidation has gained considerable attention as an efficient and environmentally friendly alternative (Guo et al. 2024). Although bismuth oxycarbonate (Bi₂O₂CO₃) is a promising photocatalyst, its wide band gap limits visible-light utilization (Guo et al. 2024) (Fan et al. 2019). Integrating a support material is regarded as a solution to overcome this difficulty (Lv et al. 2014). Graphitic carbon nitride (g-C₃N₄), characterized by a narrow band gap, high stability, and favorable adsorption properties, can enhance photocatalytic performance when combined with Bi₂O₂CO₃ by improving charge transfer and increasing surface area (Su et al. 2022). In addition, it has distinct structures enabling it to specifically interact with heavy metal ions. This feature allows the contaminants to accumulate on the surface of g-C_3_N_4_ based composites.

This study presents the first hydrothermal synthesis of a CFA/g-C_3_N_4_/Bi_2_O_2_CO_3_ composite for Pb^2^⁺ adsorption and reuse of the Pb-loaded material in RhB degradation and latent fingerprint detection. Central composite design optimization yielded a maximum Pb^2^⁺ capacity of 60.29 mg g⁻^1^. The composite exhibits dual functionality as an adsorbent and reusable photocatalyst for environmental remediation. It further supports United Nations Sustainable Development Goals (SDGs) 3, 6, 9, 12, and 15 through simultaneous heavy metal/dye remediation, coal fly ash valorization, and circular reuse of spent adsorbent.

## Materials and methods

### Materials

Bismuth nitrate pentahydrate (Bi(NO_3_)_2_.5H_2_O) (98%), lead nitrate (Pb(NO_3_)_2_) (98%), ethanol (EtOH) (99.9%), Propan-2-ol (99.5%), and methanol (MeOH) (99.9%) were purchased from Sigma-Aldrich, South Africa. Silver nitrate (AgNO_3_) extrapure AR (99.9%), L-Ascorbic acid (≥ 99%), sodium hydroxide (NaOH) and urea were purchased from ACE chem, South Africa. EDTA disodium salt (99–101%), rhodamine B (RhB) (99.8%), and polyethylene glycerol (EG) were supplied from Sisco Research Laboratories Pvt. Ltd, India).

### *Preparation of CFA/g-C*_*3*_*N*_*4*_*/Bi*_*2*_*O*_*2*_*CO*_*3*_

Graphitic carbon nitride (g-C_3_N_4_) was synthesized by the pyrolysis of urea at high temperatures. 5 g of urea were placed in an alumina crucible with a lid and placed in a furnace at 550 ℃ for 2 h with a heating rate of 5 ℃/min in an N_2_ atmosphere. After removing the kiln from the furnace cavity, a natural cooling process was done. The collected yellowish powder was treated with enough EtOH and then subjected to ultrasonic vibration by an ultrasonic cleaner bath (Scietech, South Africa) for 24 h to transform into 2D-g-C_3_N_4_.

**Scheme 1:**
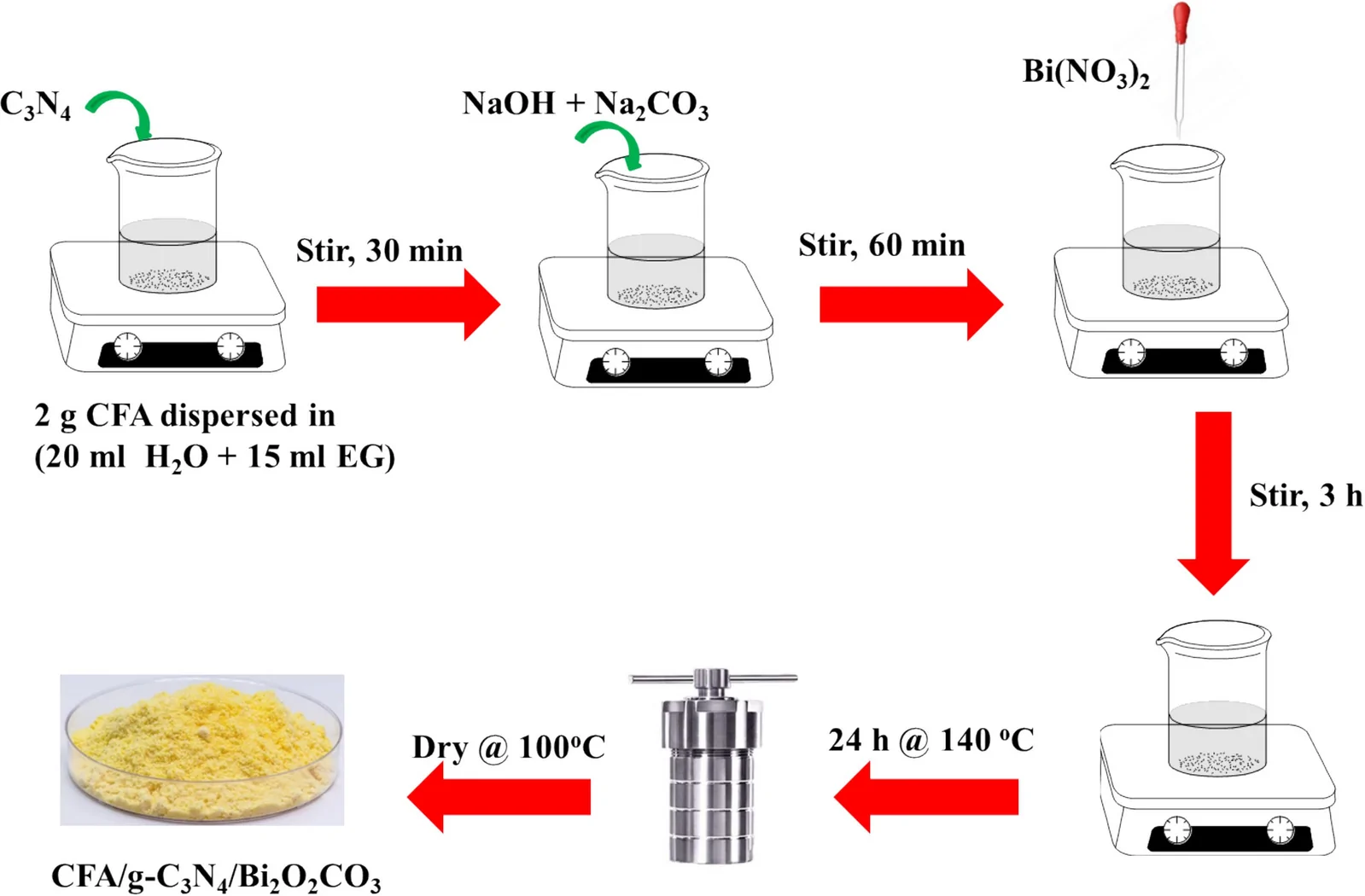
Prepartion of CFA/g-C_3_N_4_/Bi_2_O_2_CO_3_ composite

For the preparation of the CFA/g-C_3_N_4_/Bi_2_O_2_CO_3_ composite, 2 g of CFA and 0.8 g of g-C_3_N_4_ were mixed in 20 mL of deionized water and 15 mL of ethylene glycol (EG) solution and subjected to magnetic stirring with a high rotating speed for 30 min. An aqueous solution containing 15 mmol NaOH and 1 mmol Na_2_CO_3_ was introduced into the solution under vigorous stirring for 1 h. Afterwards, 5 mmol of Bi(NO_3_)_2_.5H_2_O dissolved in 5 mL deionized water was gradually added to the above sol and stirred for another 3 h at room temperature. Then, the mixture was transferred into a Teflon-lined autoclave and heated at 140 °C for 24 h in an oven (Labotec EcoTherm, South Africa). The obtained precipitate was separated by centrifugation, washed with absolute ethanol, and then dried at 100 °C overnight (Scheme 1).

### Characterization techniques

The functional groups on the as-prepared samples surfaces were determined by Fourier transform infrared spectroscope (FTIR 750, Magna, USA). For each sample, the spectra were recorded in the range of 500–4500 cm^−1^ with a resolution of 4 cm^−1^. The crystalline phases of as-prepared materials were identified by X-ray diffraction (XRD, a Philips PAnalytical Xpert PRO) under wide-angle (5–100°) scanning range at scanning rate 160 s/2θ and Cu Kα radiation (λ = 1.540 56 Å). The surface morphology of the as-prepared samples was observed with a Thermo Scientific scanning electron microscope (SEM) **(**model 6658A-1NUS-SN, USA), equipped with energy-dispersive X-ray spectroscopy (Oxford, UK).

The surface area of the as-prepared samples were measured by a Micrometrics ASAI 2020 Surface Area and Porosity Analyzer (USA). The sample’s surface area and average pore diameter were determined according to the Brunauer–Emmett–Teller (BET) and BJH methods. The material’s thermal stability was studied by a thermogravimetric analyser (TG, HITACHI STA7200RV, Japan).

The surface elemental composition was analyzed by X-ray photoelectron spectroscopy (XPS) with ultrahigh vacuum a Kratos Axis Ultra device (Kratos, UK) equipped with a monochromatic using Al Kα (1486.6 eV) as a radiation source.

Ultraviolet–visible diffuse reflectance (DRS-UV–vis) with BaSO_4_ as a reflectance standard was used to estimate the band gap energy of the as-prepared samples (UV-1208 model, Shimadzu, Japan). Photoluminescence spectroscopy (PL) (Perkin Elmer, precisely, South Africa) was used to assess the charge recombination rates of the as-prepared samples using λ_exc_ = 360 nm laser as an excitation source.

To evaluate the charge on the surface of the CFA/g-C_3_N_4_/Bi_2_O_2_CO_3_ material, the zeta potential as a function of pH was determined by a Malvern Zetasizer Nano ZS 90 (Zeta potential, ZENN 3600, UK). Using Inductively coupled plasma optical emission spectroscopy (ICP-OES) ((ICP –OES, Spectro Arcos, model Arcos FH512—Germany), the concentration of Pb(II) in solution was quantified.

The photoelectrochemical activity of the as-prepared CFA/g-C_3_N_4_/Bi_2_O_2_CO_3_ and CFA/g-C_3_N_4_/Bi_2_O_2_CO_3_@Pb^2**+**^ composites were studied on an Autolab PGSTAT204 (Netherlands) with a three-electrode system; an as-prepared samples coated on a fluorine dope titanium dioxide (FTO) glass, a platinum mesh, and Ag/AgCl (sat. KCl) are used as a working, counter, and a reference electrode, respectively. The working electrode was dipped in the 0.1 M Na_2_SO_4_ solution, and illuminated by a 100 W Xe lamp.

### *Created design and batch adsorption experiments of removal Pb*.^*2*+^*by CFA/g-C*_*3*_*N*_*4*_*/Bi*_*2*_*O*_*2*_*CO*_*3*_

#### Created design

Response surface methodology (RSM) was utilized to evaluate the relationship between % Pb^2+^ removal as an output response with contact time (32–217.5 min) (A), CFA/g-C_3_N_4_/Bi_2_O_2_CO_3_ dosage (3.18–36.82 mg) (B), and pH (1.64–8.36) (C) as independent variables. Twenty sets of experiments (3 center points and 17 factorial points) were performed based on a Central Composite Design (CCD), as shown in Table S1 (Supplementary data).

The relationship between the dependent and independent variables is expressed by a second order response surface model, as illustrated in Eq. (1) (Reghioua et al. 2024):

1 $$Y={\beta}_{0}+\sum_{i=1}^{k}{\beta}_{i}{x}_{i}+\sum_{i=1}^{k}{\beta}_{ii}{x}_{i}^{2}+\sum_{1\le i\le j}^{k}{\beta}_{ij}{x}_{i}{x}_{j}$$

Herein, Y is the response, β_0_ is a constant coefficient, x_i_ and x_j_ denote the factors (i and j range from 1 to k). β_j_, β_jj_, and β_ij_ represent the interaction coefficients of linear, quadratic and the second order terms, respectively; k denote the total number independent variables being studied (k = 3). Regression analysis and data processing was accomplished using Design Expert V.7.0. Analysis of variance (ANOVA) was employed for estimating the goodness of the model, and the mean parameters and their interaction.

#### Batch adsorption experiments

Batch adsorption experiments verified the adsorbent’s ability to adsorb Pb^2+^. The adsorption kinetics experiment was performed by mixing 10 mg of the adsorbent with 10 mL of a Pb^2+^ solution (50 mg L^−1^) at pH 4.12. The samples were taken at a specific time interval (5 min–120 min). Afterwards, an ICP-OES was utilized to quantify the residual concentration of Pb^2+^; and the adsorption capacity of the as-prepared sample at time t (q_t_(mg g^−1^)) was determined using Eq. (2):

2 $${\mathrm{q}}_{\mathrm{t}}\hspace{0.17em}=\hspace{0.17em}({\mathrm{C}}_{\mathrm{o}}-{\mathrm{C}}_{\mathrm{t}})\mathrm{V}/\mathrm{m}$$

where C_o_ (mg L^−1^) is the initial Pb^2+^ concentration, C_t_ (mg L^−1^) is the Pb^2+^ concentration at time t (min), V is the volume of solution in L, and m is is the adsorbent mass in grams.

A similar experiment was carried out to study the Pb^2+^ adsorption isotherm. The experiment was performed with the initial metal concentrations ranging from 10 to 360 mg L^−1^ at pH 4.12. The solution was shaken at 250 rpm for 2 h at 25 °C. The experiments were carried out in triplicate, and the equilibrium adsorption capacity of Pb^2+^ (q_e_(mg g^−1^)) was calculated using Eq. (3):

3 $${\mathrm{q}}_{\mathrm{e}}\hspace{0.17em}=\hspace{0.17em}({\mathrm{C}}_{\mathrm{o}}-{\mathrm{C}}_{\mathrm{e}})\mathrm{V}/\mathrm{m}$$

where C_e_ is the equilibrium concentration of Pb^2+^.

For adsorption thermodynamics, 10 mg of adsorbent was added to 10 mL of a Pb^2+^ solution at a concentration of 50 mg L^−1^. The experimental temperature was set at 25 °C, 35 °C, and 45 °C. The solution pH was adjusted by 0.1 M HCl/NaOH to examine the pH’s effect on adsorbent performance.

### *Reusability of the lead-loaded spent adsorbent (CFA/g-C*_*3*_*N*_*4*_*/Bi*_*2*_*O*_*2*_*CO*_*3*_*@Pb*^*2*+^*) in photocatalytic degradation of Rhodamine B and created design experiments*

The photocatalytic degradation of rhodamine B (RhB) by the lead-loaded spent adsorbent (CFA/g-C_3_N_4_/Bi_2_O_2_CO_3_@Pb^2+^) and CFA/g-C_3_N_4_/Bi_2_O_2_CO_3_ composite was assessed at room temperature, and a 250 HW xenon mercury lamp was used as a visible light source. The experiments were conducted in a Pyrex top irradiation reaction vessel (Lelesil Innovative Systems, India). The experiments were carried out with the CFA/g-C_3_N_4_/Bi_2_O_2_CO_3_ or CFA/g-C_3_N_4_/Bi_2_O_2_CO_3_@Pb^2+^ (0.05 g) dispersed into RhB aqueous solutions (50 mL, with concentration of 5 mg L^−1^ and an initial pH of 3.05). Before the catalytic test, the mixture was stirred in the dark for 1 h to attain adsorption–desorption equilibrium. Then, the light was turned on to begin the photocatalytic reaction. An aliquot of 5 mL of the reaction solution was taken at a specific time intervals and filtered. The concentration of residual RhB in the filtrate was quantified by an ultraviolet–visible spectrophotometer (Perkin Elmer double beams spectrophotometer, Germany) with the characteristic absorption peak of 554 nm. The removal efficiency of RhB dye was calculated by Eq. (4).

4 $$\mathrm{R}\mathrm{e}\mathrm{m}\mathrm{o}\mathrm{v}\mathrm{a}\mathrm{l} \mathrm{e}\mathrm{f}\mathrm{f}\mathrm{e}\mathrm{c}\mathrm{i}\mathrm{e}\mathrm{n}\mathrm{c}\mathrm{y} \left(\mathrm{\%}\right)=\frac{{\mathrm{C}}_{0}-{\mathrm{C}}_{\mathrm{t}}}{{\mathrm{C}}_{0}}\times 100$$

where C_0_ represents the initial concentration of RhB, and C_t_ denotes the concentration after a certain time of photo-catalysis, all in mg L^−1^.

The free radical trapping experiment was performed by adding 25 mg of the CFA/g-C_3_N_4_/Bi_2_O_2_CO_3_@Pb^2+^ composite to 25 ml of a RhB solution at a concentration of 5 mg/L. Propan-2-ol (1mM), L-Ascorbic acid (1mM), Ethylenediaminetetraacetic acid (EDTA-2Na, 1mM) and Silver Nitrate (1mM) were used as scavengers to inhibit hydroxyl radicals (OH^•^), superoxide radicals(•O_2_^−^), holes (*h*^+^) and electrons (e^−^), respectively (Abedini et al. 2021).

In the recycling stability experiment, the the CFA/g-C_3_N_4_/Bi_2_O_2_CO_3_@Pb^2+^ composite was separated from the reaction solution by centrifugation. The recovered sample was washed thoroughly with ethanol until no colour on the surface of the sample was observed; then dried at 60 °C in an oven to reuse in the next cycle.

### Central Composite Design (CCD)

In the optimization the photocatalytic degradation of RhB by the CFA/g-C_3_N_4_/Bi_2_O_2_CO_3_@Pb^2+^ composite, we used CCD experimental design to optimize three dependent variables: (A) contact time (5–375 min), (B) amount of photocatalyst (3.07–61.9 mg), and (C) pH of the solution (1.3–9.7) on the photodegradation activity of RhB. Photodegradation activity of RhB was used as the independent variable. The CCD generated 20 experimental runs, and the obtained matrix is listed in Table S2 (Supplementary data).

### *Reusability of the CFA/g-C*_*3*_*N*_*4*_*/Bi*_*2*_*O*_*2*_*CO*_*3*_*@Pb*^*2*+^*composite in latent fingerprint application*

The powder dusting method (PDM) with a recyclable CFA/g-C_3_N_4_/Bi_2_O_2_CO_3_@Pb^2+^ composite identifying powder was examined for latent fingerprint application. The donor’s hands were first cleaned and dried before taking fingerprints. After applying the fingers cleanly to the noses and foreheads, the thumb impression was then put on various surfaces. The CFA/g-C_3_N_4_/Bi_2_O_2_CO_3_@Pb^2+^ labelling powder was then applied to the fingerprint, and a squirrel brush was used to remove any excess powder. The fingerprint photos were captured under daylight conditions using a smartphone with a high-resolution camera.

## Result and discussion

### *Structural characteristics of CFA/g-C*_*3*_*N*_*4*_*/Bi*_*2*_*O*_*2*_*CO*_*3*_* composite adsorbent*

#### X-ray diffraction analysis

**Fig. 1:**
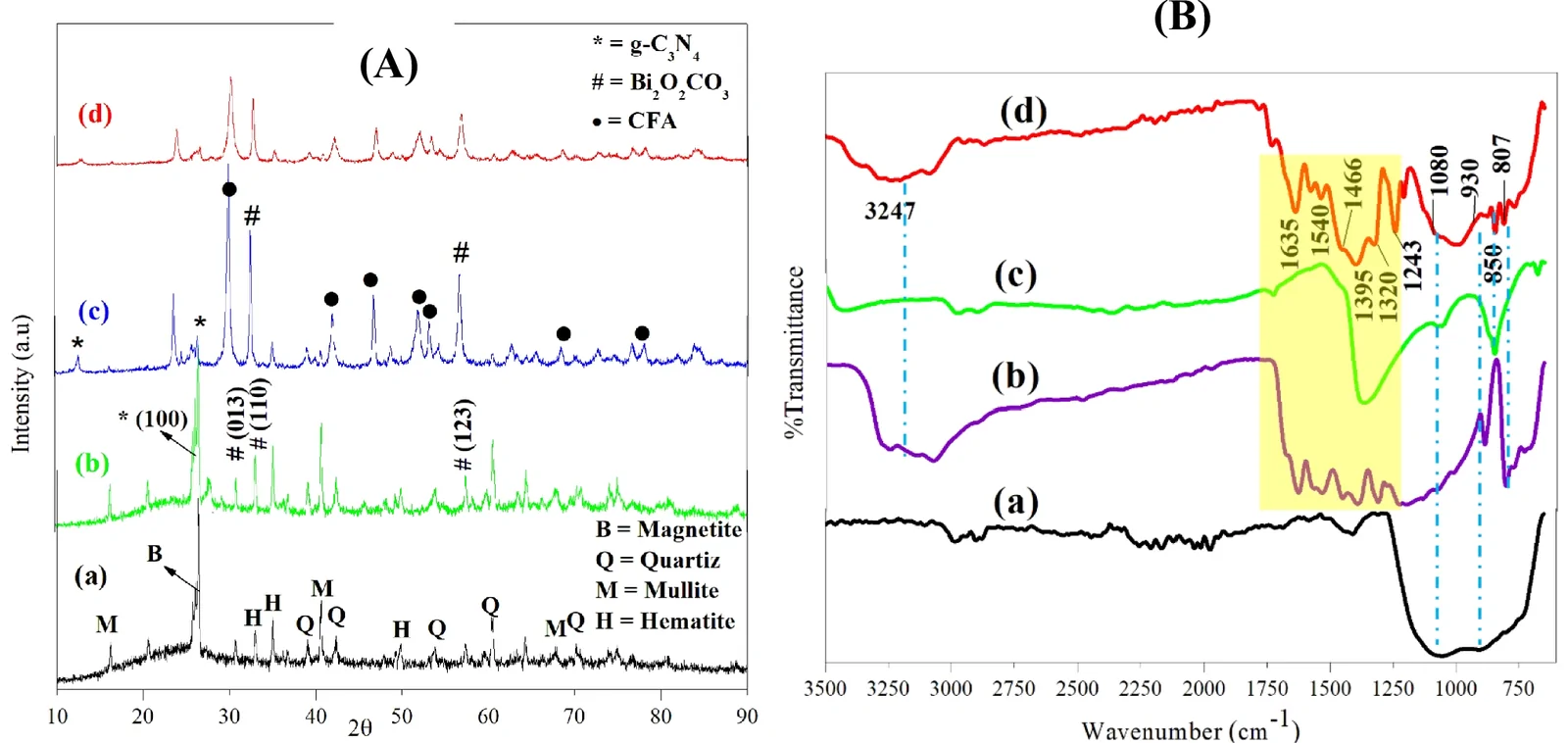
(**A**) XRD patterns of (**a**) CFA (**b**) g-C_3_N_4_/Bi_2_O_2_CO_3_ (c) CFA/g-C_3_N_4_/Bi_2_O_2_CO_3_ and (**d**) CFA/g-C_3_N_4_/Bi_2_O_2_CO_3_@Pb^2+^, (**B**) FT-IR spectra for: (**a**) CFA, (b) g-C_3_N_4_, (c) Bi_2_O_2_CO_3_ and (d) CFA/g-C_3_N_4_/Bi_2_O_2_CO_3_

The X-ray diffraction (XRD) patterns of CFA, g-C_3_N_4_/Bi_2_O_2_CO_3_, CFA/g-C_3_N_4_/Bi_2_O_2_CO_3_ and the CFA/g-C_3_N_4_/Bi_2_O_2_CO_3_@Pb^2+^ composites are displayed in Fig. 1A. As shown in Fig. 1A(a), the CFA exhibited the diffraction patterns at 16.17°, 20.48°, 26.74°, 33.13°, 34.98°, 40.77°, 50.09°, and 62.62° are attributed to crystalline phases of mullite, magnetite, hematite and quartz (Umejuru et al. 2021). The diffraction pattern of g-C_3_N_4_/Bi_2_O_2_CO_3_ showed distinct diffraction peaks at 13.04° and 26.97° which correspond to the (1 0 0) and (0 0 2) graphitic structure of g-C_3_N_4_ (Fig. 1A(b)) (JCPDS No. 87e1526) (Balu et al. 2020). In addition, the 2θ values of 30.85°, 33.35° and 57.32° assigned to the (0 1 3), (1 1 0) and (1 2 3) crystalline facets of Bi_2_O_2_CO_3_, respectively (JCPDS NO. 04–014–4950) (Li et al. 2021). The coexistence of the characteristic peaks of CFA, g-C_3_N_4_ and Bi_2_O_2_CO_3_ indicated the successful formation of the as-synthesized heterojunction composite material (Fig. 1A(c)). Furthermore, the sharper diffraction peaks prove that the as-synthesized CFA/g-C_3_N_4_/Bi_2_O_2_CO_3_ composite has higher structural crystallinity. The X-ray diffraction (XRD) pattern of the CFA/g-C_3_N_4_/Bi_2_O_2_CO_3_@Pb^2+^ composite exhibited a similar diffraction pattern with a decrease in peak intensities after Pb^2+^ ion adsorption (Fig. 1A(d)). The reduction in peak intensities reveals the strong interaction between Pb^2+^ ions and the negatively charged surface of the as-synthesized composite material.

#### Fourier transform infrared (FT-IR) study

FT-IR spectroscopy further provided evidence of the formation of the as-synthesized composite material. From Fig. 1B, the pristine CFA shows two prominent bands at 1395 cm^−1^ and 1080 cm^−1^ assigned to the symmetric and asymmetric stretching vibrations of the Al–O–Si bond; whereas the band at 930 cm^−1^ is attributed to the symmetric stretching of the Si–OH group (Gadore & Ahmaruzzaman 2021; Umejuru et al. 2021). For pure g-C_3_N_4_, the broader band centred at 3247 cm^−1^ is assigned to the aromatic ring’s symmetric stretching modes of N–H. The signals at 1635 cm^−1^, 1540 cm^−1^, 1466 cm^−1^, 1320 cm^−1^ and 1243 cm^−1^ correspond to the stretching vibrations of C = N and C-N of heterocycles units, while the breathing vibration of triazine rings is seen at 807 cm^−1^ (Zhang et al. 2022). The pure Bi_2_O_2_CO_3_ shows two characteristic bands at 1395 cm^−1^ and 850 cm^−1^ (asymmetric and bending vibrations of the CO_3_^2−^ group). For the CFA/g-C_3_N_4_/Bi_2_O_2_CO_3_ composite, the existence of its components peaks in the spectrum demonstrates the successful incorporation of these components in the matrix.

#### Thermal analysis

Thermogravimetric analysis (TGA) and differential thermal analysis (DTA) analysis were utilized to examine the thermal stability of CFA/g-C_3_N_4_/Bi_2_O_2_CO_3_ composite; the results are provided in Fig. S1A. In the first and second stages, there is slight a weight loss (0.9%) between 50 and 120 °C and 370 to 420 °C (2.1%), which resulted from the evaporation of bound water and residual volatile organic compounds (acetone, ethanol and DMF, etc.) in the material. In the third stage (425–560 °C), Bi_2_O_2_CO_3_ in the adsorbent converted into Bi_2_O_3_, resulting in a remarkable weight loss (13.6%). The analysis of the DTA curve in Fig. S1A revealed that the decomposition of the carbonate group is an exothermic reaction that occurs at 466.9 °C. It is also evident that the decomposition temperature of the carbonate group for the Bi_2_O_2_CO_3_ in the composite is higher than that of pure Bi_2_O_2_CO_3_ (356 °C) caused by the strong interaction of the heterojunction phases, thus confirming the formation of CFA/g-C_3_N_4_/Bi_2_O_2_CO_3_ composite adsorbent (Lopes et al. 2018). At above 600 °C, the remaining weight of CFA/g-C_3_N_4_/Bi_2_O_2_CO_3_ is 83.4%, This indicates that the CFA/g-C_3_N_4_/Bi_2_O_2_CO_3_ adsorbent has strong thermal stability and support for future wastewater applications.

#### Brurnauer-Emmett-Teller (BET) characterization

The nitrogen adsorption–desorption isotherms of CFA/g-C_3_N_4_/Bi_2_O_2_CO_3_ composite are shown in Fig. S1B. It can be found that the as-synthesized composite exhibits the type IV isotherm with H3 hysteresis loop model and average pore diameter of 19.091 nm, which indicated the composite possesses a mesoporous structure and slit-shaped pores inside the adsorbent (Li et al. 2023)*.* The measured BET surface area of CFA/g-C_3_N_4_/Bi_2_O_2_CO_3_ composite was estimated as (10.845 m^2^/g) which is much larger than that of CFA (6.075 m^2^/g) reported elsewhere (Umejuru et al. 2021). The high specific surface area of CFA/g-C_3_N_4_/Bi_2_O_2_CO_3_ composite is desirable for the removal of Pb^2+^ ions and photocatalytic degradation of RhB.

#### Surface morphology

**Fig. 2:**
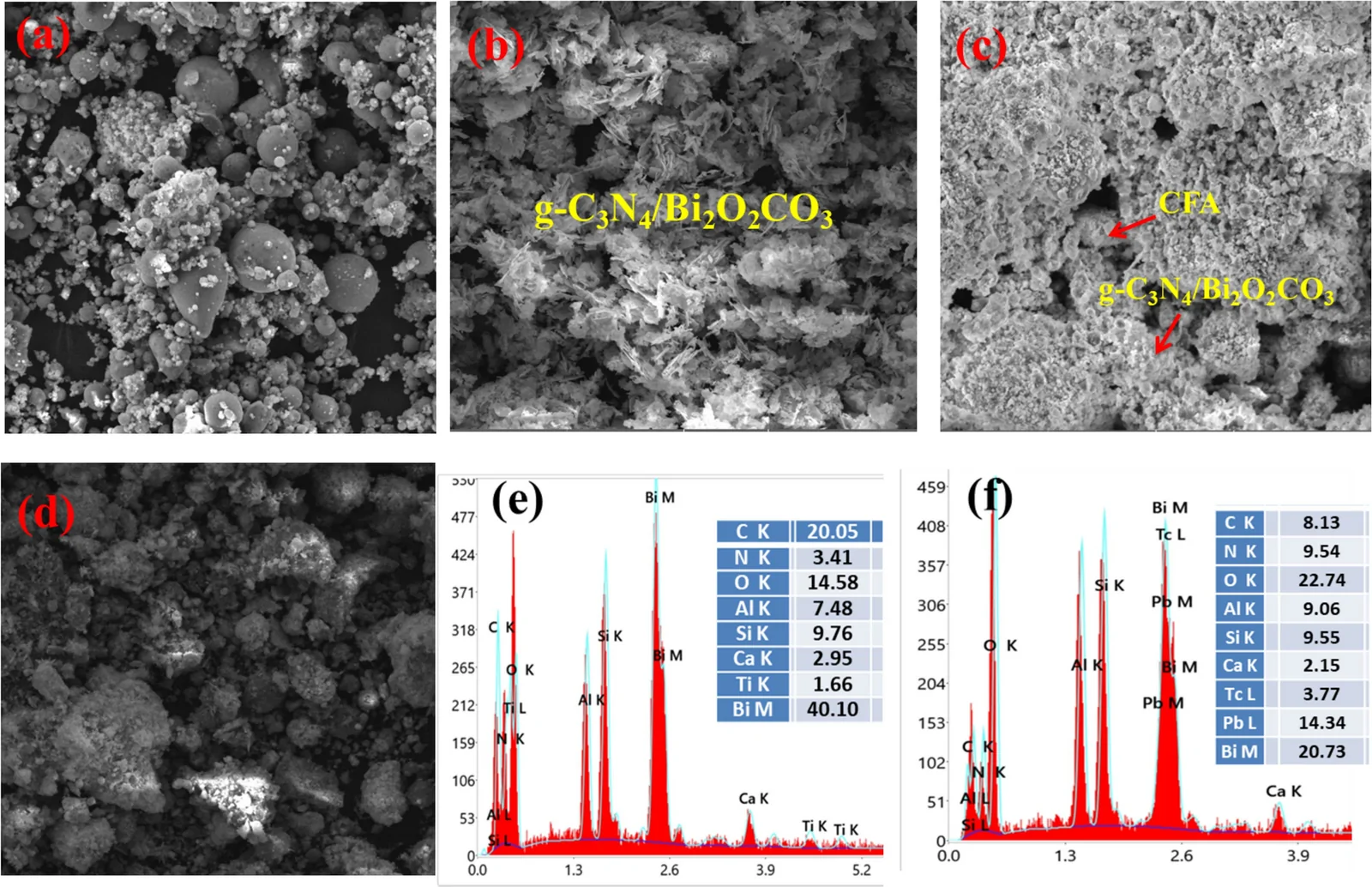
(**a**–**f**), (**a**) SEM image of CFA, (**b**) g-C_3_N_4_/Bi_2_O_2_CO_3_, (**c**) CFA/g-C_3_N_4_/Bi_2_O_2_CO_3_, (**d**) CFA/g-C_3_N_4_/Bi_2_O_2_CO_3_@Pb^2+^, EDX of CFA/g-C_3_N_4_/Bi_2_O_2_CO_3_ composite (**e**) before and (**f**) after adsorption

The morphology and structure of the CFA, g-C_3_N_4_/Bi_2_O_2_CO_3_ and CFA/g-C_3_N_4_/Bi_2_O_2_CO_3_ composite before and after adsorption were examined by SEM and EDX (Fig. 2a–f). According to the SEM micrograph of pristine CFA shown in Fig. 2a, the CFA presents spherical particles with a glossy surface and a hollow appearance. The micrograph of the g-C_3_N_4_/Bi_2_O_2_CO_3_ composite (Fig. 2b) reveals that the Bi_2_O_2_CO_3_ crystals are distributed on the stacking layers of g-C_3_N_4_, composing small and large netlike sheets. Figure 2c shows that CFA/g-C_3_N_4_/Bi_2_O_2_CO_3_ composite adsorbent has thick layers, probably formed by the agglomeration of g-C_3_N_4_ and Bi_2_O_2_CO_3_ particles onto the cenospheres surface of CFA. It is also evident from Fig. 2c that the CFA/g-C_3_N_4_/Bi_2_O_2_CO_3_ composite adsorbent has an irregular and rough surface, making it appropriate for Pb^2+^ ions removal (Al-Ahmed et al. 2024). Figure 2d displays the SEM image of the CFA/g-C_3_N_4_/Bi_2_O_2_CO_3_ composite after Pb^2+^ ions adsorption. Compared with the CFA/g-C_3_N_4_/Bi_2_O_2_CO_3_ composite adsorbent, it can be found that there was no significant morphological structure change after adsorption. This suggests that the CFA/g-C_3_N_4_/Bi_2_O_2_CO_3_ composite adsorbent is stable, which is consistent with the XRD and later EDX results.

Figure 2(e–f) provides the EDX elemental composition of the CFA/g-C_3_N_4_/Bi_2_O_2_CO_3_ composite before and after adsorption. Observations from Fig. 2d reveals the presence of the elements C, Al, and Si while Ti and Ca exist at minute percentages. It is also evident from the EDX in Fig. 2e that the N, O and Bi elements coexist in an as-synthesized composite, which indicates the successful preparation of CFA/g-C_3_N_4_/Bi_2_O_2_CO_3_ composite adsorbent. After Pb^2+^ adsorption (Fig. 2f), similar elements were scattered on the surface of CFA/g-C_3_N_4_/Bi_2_O_2_CO_3_ composite adsorbent, and the appearance of Pb^2+^ indicated the successful adsorption of Pb^2+^ ions.

#### XPS analysis

X-ray photoelectron spectroscopy was utilized to further study the chemical composition of CFA/g-C_3_N_4_/Bi_2_O_2_CO_3_ composite adsorbent. Figure 2S (Supplementary data) displays the survey of CFA/g-C_3_N_4_/Bi_2_O_2_CO_3_. In the spectrum, the peaks at 282.6 eV (C1s), 396.3 (N1s), 528.6 eV (O1s), 439.6 eV (Bi^3+^ 4d_5/2_), 463.5 eV (Bi^3+^ 4d_3/2_), 157.3 eV (Bi^3+^ 4f_7/2_) and 162.5 eV (Bi^3+^4f_5/2_) prove the CFA/g-C_3_N_4_/Bi_2_O_2_CO_3_ composite adsorbent was successfully prepared (Xue et al. 2022; Zhang et al. 2022).

### Batch adsorption

#### Statistical analysis and optimization by central composite design

The analysis of variance (ANOVA) was performed to calculate the *p*-value of each term of the model, and the obtained results are illustrated in Table S3 (Supplementary data). Terms with *p*-values less than 0.05 were considered to be significant. Based on the p-values of the terms in the regression model, (*p* < 0.05), the independent variables that affected the Pb^2+^ removal efficiency were: Contact time (A), CFA/g-C_3_N/Bi_2_O_2_CO_3_ dosage (B), Solution pH (C), CFA/g-C_3_N/Bi_2_O_2_CO_3_ dosage*Solution pH (BC), (Contact time)^2^ (A^2^), (CFA/g-C_3_N_4_/Bi_2_O_2_CO_3_ dosage)^2^ (B^2^), (Solution pH) 2 (C2) and Contact time*(CFA/g-C_3_N_4_/Bi_2_O_2_CO_3_ dosage)^2^ (AB^2^). From the data in Table S3, the response (% of removal Pb^2+^) as a function of independent variables was obtained and represented by the following expression (Eq. (5)):

5 $$\mathrm{\%} \mathrm{o}\mathrm{f} \mathrm{r}\mathrm{e}\mathrm{m}\mathrm{o}\mathrm{v}\mathrm{a}\mathrm{l} {\mathrm{P}\mathrm{b}}^{2}+ \hspace{0.17em}=\hspace{0.17em}\hspace{0.17em}+\hspace{0.17em}97.52\hspace{0.17em}+\hspace{0.17em}14.02\mathrm{A}\hspace{0.17em}+\hspace{0.17em}17.40 \mathrm{B}\hspace{0.17em}+\hspace{0.17em}21.37\mathrm{C}\hspace{0.17em}+\hspace{0.17em}0.020\mathrm{A}\mathrm{B}-0.93\mathrm{A}\mathrm{C}-18.35\mathrm{B}\mathrm{C}-6.{19\mathrm{A}}^{2}-8.07{\mathrm{B}}^{2}-13.34{\mathrm{C}}^{2}-12.39\mathrm{A} {\mathrm{B}}^{2}$$

The model’s validity was justified through the analysis of residue which enables calculating the coefficient of determination (*R*^2^). With an **R**^2^ value of 0.9850, about 98.5% of the variability in the response variable was explained by the model, thus signifying a robust agreement between the actual and predicted values. The adjusted *R*^2^ of 0.9683 suggests that the 96.83% predictor variables considerably contribute to the model’s explanatory power. Additionally, the regression model for Pb^2+^ removal is statistically significant with p value lower than 0.05, at 95% confidence. Meanwhile, the lack-of-fit (LOF) of the p value 0.0827 reflects that LOF is statically insignificant with regard to pure error at 95% confidence level. Furthermore, the CV% and Adequate Precision, at 6.96% and 23.081, respectively, denote a desirable signal-to-noise ratio, demonstrating the model’s suitability for predicting the variable within the defined experiment domain (Al-Ahmed et al. 2024; Xiao et al. 2017).

**Fig. 3:**
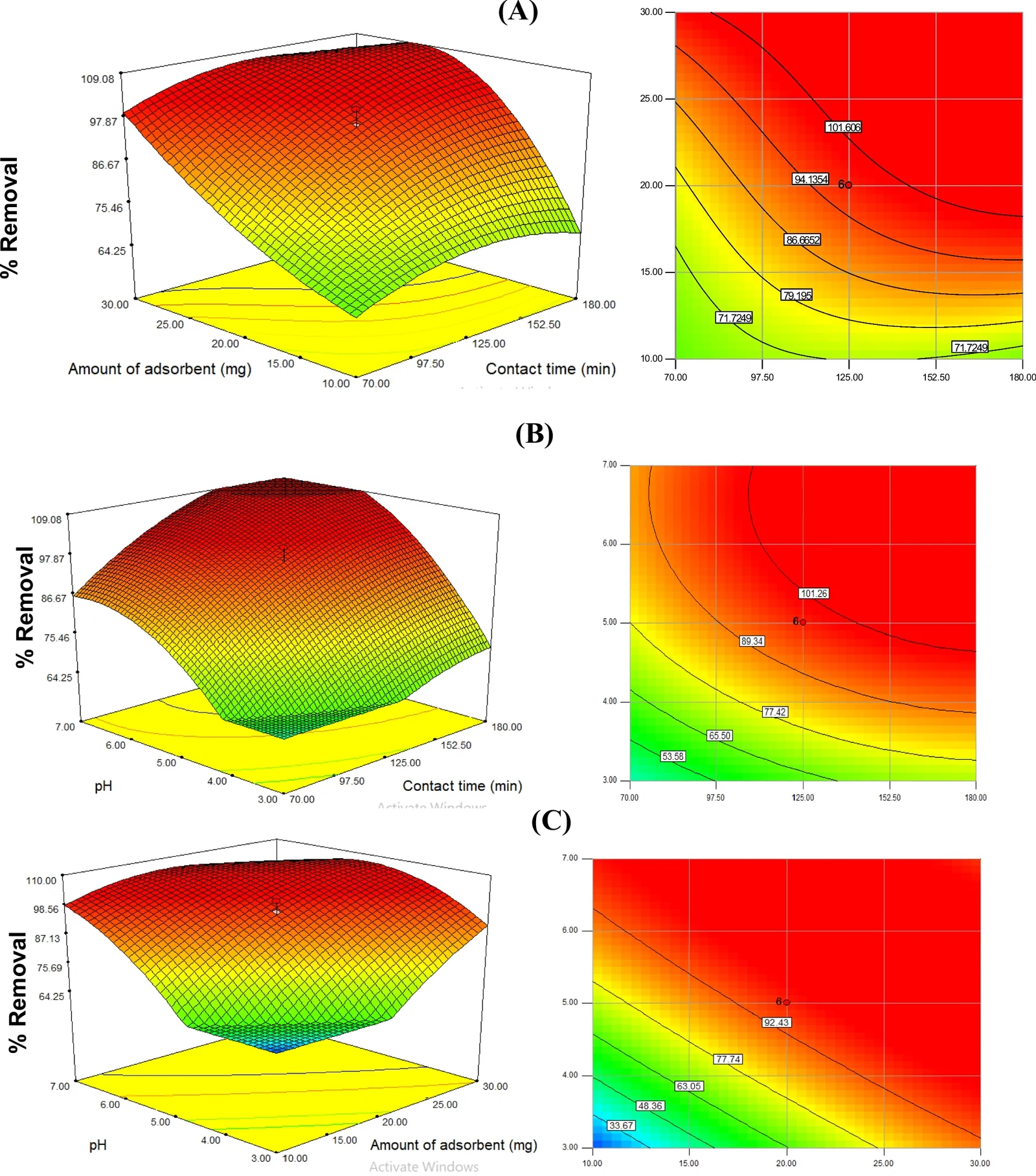
Surface plots (left panel) and contour plots (right panel) as a function of (**a**) amount of adsorbent and contact time, (**B**) Initial pH and contact time, and (**C**) Initial pH and amount of adsorbent in Pb^2+^ ion removal by CFA/g-C_3_N_4_/Bi_2_O_2_CO_3_ composite

The model’s adequacy was also checked by comparing the distribution of predicted and experimental values of the response (Fig. 3SA in supplementary data). The points are statistically distributed around the line, which affirms the model’s proficiency in correlating between the experimental and predicted values. Moreover, the normal plot vs residual (Fig. 3SB in supplementary data) gives a straight line, emphasizing the premise of normality (Al-Ahmed et al. 2024). In summary, these results collectively demonstrate the model’s robustness and statically significant for predicting the response variable and thus it will be further utilized in statistical analysis.

Figure 3A–C shows a detailed evaluation of the interaction between important process variables including contact time (min), amount of adsorbent (mg) and initial solution pH, and their corresponding contour plot to attain the highest Pb^2+^ removal percentage by CFA/g-C_3_N_4_/Bi_2_O_2_CO_3_. Figure 3A shows that the amount of adsorbent and contact time have a considerable impact on Pb^2+^ removal, with the addition of the adsorbent and longer contact times resulting in enhanced efficiency. This phenomenon implies that the adsorption capability of CFA/g-C_3_N_4_/Bi_2_O_2_CO_3_ is directly related to the available adsorption sites and the time to occupy the active sites.

Figure 3B displays the relationship between contact time and initial solution pH on the removal efficiency of Pb^2+^. Notably, increasing solution pH from 3 to 5, particularly at a longer interaction time enhances the Pb^2+^ removal efficiency. At pH 5.5, the presence of Pb^2+^ and PbOH^+^ species facilitated attractive interactions between the negatively adsorbent surface and Pb^2+^ metal ions, resulting in increasing adsorption efficiency (> 100%). Finally, Fig. 3C displays the interaction between initial pH and the amount of adsorbent, revealing that the amount of adsorbent has a significant impact on Pb^2+^ removal, whereas pH plays a key role in the adsorption process.

To determine the best operating parameters for removing Pb^2+^ from an aqueous solution using the CFA/g-C_3_N_4_/Bi_2_O_2_CO_3_, verification experiments were carried out under the optimum conditions (Supplementary Table S4). As shown, the optimal conditions for removing Pb^2+^ were a contact time of 107.6 min, an adsorbent dosage of 24.35 mg, and a pH of 6.62.

While the correspondingactual value of removing Pb^2+^ under the same conditions was found to be 99.99% ± 0.52 (mean ± SD of three replicates), which is close to the theoretical value (101.52%) with error margin of < 1.13. The good correlation between the theoretical and actual results implies that the CCD combined with Derringer’s desirability function could be utilized to optimize the independent variables that affect the removal of Pb^2+^.

#### Adsorption kinetics

Understanding the kinetics of adsorption is critical for wastewater treatment. As a result, the nonlinear pseudo first order, pseudo second order models and Elovich were used to assess the adsorption kinetic behavior that describes the Pb^2+^ sorption system on CFA/g-C_3_N_4_/Bi_2_O_2_CO_3_ (Sachit 2024).The nonlinear coefficient of determination (*R*^2^) and relative function F_error_ are used to validate the models. The earlier statistical indicies showed the variations between the experimental and the predicted values of the Pb.^2+^ ion adsorption capacity of the adsorbent, which are provided by Eq. (6) (Ahamed et al. 2013)

6 $${F}_{error}=\sqrt{\sum_{i}^{p}{\frac{\left({q}_{imodel}-{q}_{texperimental}\right)}{{q}_{texpermental}}}^{2}}.(\frac{1}{p-1})$$

where q_t model_ is each value of q_tpredicted_ by the fitted model and q_texperimental_ is each value of q_tmeasured_ experimentally, and p is the number of experiments performed.

The nonlinear equations of these three kinetic models are shown (Fig. S4A, supplementary data), and the resultant kinetic parameters are provided in Table S5 (supplementary data). The pseudo-second-order equation accurately represents the adsorption of Pb^2+^ ion metals onto CFA/g-C_3_N_4_/Bi_2_O_2_CO_3_, with higher coefficient of determination (*R*^2^ = 0.9969) and smaller relative average error (F_error_ < 0.61). These results imply that Pb^2^⁺ adsorption onto the CFA/g-C_3_N_4_/Bi_2_O_2_CO_3_ surface is primarily chemisorption, which involves the exchange or sharing of electrons between the Pb^2^⁺ ions and the functional groups on the adsorbent surface. In addition, the relative high coefficient of determination (*R*^2^ > 0.95) and the lowest relative average error (F_error_ < 0.096) shows that the experimental data conforms Elovich equation as well. This suggests that the nature of adsorption Pb^2+^ ion on heterogeneous surface is chemisorption. The efficacy of the adsorption interaction between the adsorbents and the Pb^2+^ ion is specifically indicated by the greater values of the initial adsorption rate (α) relative to the desorption constant (β) (Abdulkareem et al. 2019).

To determine the rate-controlling step involved in the sorption process, the intra-particle diffusion equation was studied. Fig. S4B (supplementary data) depicts the absorption of Pb^2+^ ion metals vs t^1/2^ over the CFA/g-C_3_N_4_/Bi_2_O_2_CO_3_ adsorbent. The data points in the graphic were separated into two straight lines. The initial sharper straight segment (t_1_ = 40 min) is associated with external mass transport and ion adsorption on the external surface of CFA/g-C_3_N_4_/Bi_2_O_2_CO_3_. The second linear phase (t_2_ = 20 min) is the progressive adsorption stage, which represents intra-particle diffusion and metal ion transfer to adsorbent pores. The difference between t_1_ (40 min) and t_2_ (20 min) indicates that metal adsorption on the CFA/g-C_3_N_4_/Bi_2_O_2_CO_3_ adsorbent is controlled by external mass transport. Meanwhile, the plot’s intercept indicates the thickness of the border layer. Clearly, the intercept value (Table S5, supplementary data) is large, showing that external mass diffusion contributes more at higher initial metal ion concentrations.

#### Adsorption isotherms

Adsorption isotherms seek to understand the relationship between the quantities of solute retained at the adsorbent interface and the residual concentration of the adsorbate in the solution at equilibrium. These are is beneficial in measuring the binding behavior of adsorbate toward the adsorbent surface, which is a valuable tool for understanding the sorption mechanism (Dad et al. 2024). Additionally, the adsorption isotherm is important in determining the effectiveness of the adsorbent and the feasibility of the sorption process (Kothavale et al. 2023). The nonlinear Langmuir, Freundlich, and Temkin isotherm equations were used to describe the adsorption data for the removal of Pb^2+^ metal ions onto CFA/g-C_3_N_4_/Bi_2_O_2_CO_3_ under optimum experimental conditions (defined by DoE) (Ahamed et al. 2013; Sachit 2024).

The experimental adsorption data were fitted with the three isotherm models as indicated above (Fig. S5A), and the resulting values are shown in Table S6 (supplementary data). The Langmuir equation produced the best fit (*R*^2^ = 0.9771; F_error_ = 0.431) to experimental data, making it the most appropriate for describing adsorption. Despite the intrinsic heterogeneity of raw CFA, modification with g-C₃N₄ and Bi_2_O_2_CO_3_ produces abundant, evenly distributed surface functional groups (–NH, –NH₂, and –OH), creating adsorption sites with similar binding affinity for Pb^2^⁺ ions (Liu et al. 2018; Sachit 2024). This adsorption characteristic is consistent with previous studies on coal fly ash (CFA)-based composite adsorbents (Abd El-Fattah et al. 2026; Sopanrao & Sreedhar 2025; Sukchit et al. 2025). The Langmuir constants (K_L_) was 6.621 L g^−1^ demonstrating strong chemisorptive interactions. The change of Gibbs free energy (ΔG = − RTlnK_L_) of this system is –14.6 kJ mol⁻^1^, indicating that chemical adsorption mechanisms plays a major role (Abdulkareem et al. 2019).

It is also evident from Table S6 (supplementary data) that the value of Langmuir coefficient (K_L_) is a positive value (6.621 L g^−1^), which indicates the adsorption process is spontaneous (Anugrahwati et al. 2021). The Langmuir coefficient, K_L_, was used to calculate the separation factor constant (R_L_) of adsorbent (R_L_ = 1/(1 + K_L_C_o_)), because the values of R_L_ is commonly used to determine the degree of suitability of the adsorbent towards metal ions, Thus, when 0 < R_L_ < 1 the sorption is favorable sorption, whereas when R_L_ > 1.0 then it is unfavorable (Ahamed et al. 2013). The values of R_L_ calculated for the adsorptionf Pb^2+^ ions onto CFA/g-C_3_N_4_/Bi_2_O_2_CO_3_ were in the range between 0.0006 and 0.003, thus indicating that the adsorption process is favorable (Ahamed et al. 2013). In addition, the values of the Freundlich coefficient (K_F_) for adsorption Pb^2+^ ions on CFA/g-C_3_N_4_/Bi_2_O_2_CO_3_ is high (14.181 L g^−1^), suggesting greater ability of the adsorbent to adsorb Pb^2+^ ions (Arofah et al. 2024).

As shown in Table 1, the CFA/g-C_3_N_4_/Bi_2_O_2_CO_3_ composite exhibited competitive performance for Pb(II) removal, with a Langmuir maximum adsorption capacity of 60.29 mg g⁻^1^ under the optimized operating conditions of pH 4.12, a contact time of 120 min, an adsorbent dosage of 1.0 g L⁻^1^, and a temperature of 298 K. This capacity is more than sixfold higher than that reported for unmodified CFA (0.36–8.80 mg g⁻^1^) and considerably greater than those of low-cost adsorbents, including bentonite and red earth (8.55–10.31 mg g⁻^1^), which generally require substantially higher adsorbent dosages of 4–10 g L⁻^1^. Although advanced adsorbents, such as amino-functionalized activated carbon (T-AC, 268.61 mg g⁻^1^) and modified CFA–chitosan composite (MCFA-CS, 352.19 mg g⁻^1^), demonstrate superior adsorption capacities, their preparation commonly involves complex precursors, prolonged equilibrium times, and higher synthesis costs. These limitations may restrict their large-scale implementation and economic feasibility compared with CFA/g-C_3_N_4_/Bi_2_O_2_CO_3_. Therefore, the synthesized CFA/g-C_3_N_4_/Bi2O_2_CO_3_ composite represents a more economically favorable adsorbent for Pb^2^⁺ removal than the aforementioned materials.

**Table 1:** Comparative assessment of Pb^2+^ ions adsorption capacity of CFA/g-C_3_N_4_/Bi_2_O_2_CO_3_ composite with other cited adsorbents

Adsorbent	Time (min)	Dose (g L^−1^)	Intial conc (mg L^−1^)	Adsorption capacity (mg g^−1^)	Reference
Native natural bentonite	180	4.0	10–100	8.55	(Esmaeili & Eslami 2019)
Red earth	30	10.0	10–100	10.31	(Esmaeili & Eslami 2020)
MCFA	240	20.0	10–1000	31.38	(Astuti et al. 2021)
CFA	300	150.0	1–100	0.36	(Kuncoro & Fahmi 2013)
CFA/Fe_3_O_4_ composites	90	3.0	5–25	33.35	(Arofah et al. 2024)
CFA/GO/WO_3_ nanocomosite	60	1.3	50–300	41.51	(Umejuru et al. 2021)
zeolite A from CFA	15	6.0	50–250	8.80	(Sukchit et al. 2025)
OTAC/Mt-FA	240	4.0	NA	39.90	(Wang et al. 2025)
T-AC	240	0.5	0–210	268.61	(Liu et al. 2024)
MCFA–CS	20	1.0	100–1000	352.19	(Sopanrao & Sreedhar 2025)
CFA/g-C_3_N_4_/Bi_2_O_2_CO_3_ composite	120	1.0	10–360	60.29	This work

*MCFA*, Modified coal fly ash; *T-AC*, Amino functionalize dactivated carbon derived from coal gasifcation fine slag; *CFA-CS*, H_3_PO_4_-modified fly ash–chitosan composite; *OTAC/Mt-FA*, Montmorillonite-fly ash with octadecyltrimethylammonium chloride; *GO*, graphene oxide.

Fig. S5A depicts the nonlinear relationship between q_e_ and C_e_, and the Temkin constants are given in Table S6 (supplementary data). The relative high coefficient of determination (*R*^2^ > 0.87, F_error_ < 0.92) shows that the experimental data conforms Temkin model. As a result, the adsorption of Pb(II) ions onto the CFA/g-C_3_N_4_/Bi_2_O_2_CO_3_ can be attributed to chemisorption. This is consistent with the Langmuir isotherm findings. On the other hand, values of b (0.512 KJmol^−1^) provide additional evidence that the adsorption process of Pb(II) ions onto the CFA/g-C_3_N_4_/Bi_2_O_2_CO_3_ involves both chemisorption and physisorption (Ahamed et al. 2013).

#### Adsorption thermodynamics

The direction and character of the sorption process are known to be revealed by thermodynamic parameters such as change in free energy (ΔG°), enthalpy (ΔHº), and entropy (ΔS). The following formulae were used to determine these parameters' values (Sachit 2024).

7 $${\mathrm{logK}}_{\mathrm{C}}=\frac{\Delta \mathrm{S}}{2.303\mathrm{R}}-\frac{\Delta {\mathrm{H}}^{^\circ }}{2.303\mathrm{R}\mathrm{T}}$$

8 $$\Delta {\mathrm{G}}^{^\circ }=\Delta {\mathrm{H}}^{^\circ }-\mathrm{T}\Delta \mathrm{S}$$

T and R stand for temperature in Kelvin and the universal gas constant (8.314 J mol-1K^−1^), respectively, while K_c_ is the equilibrium coefficient. Table S7 and Fig. S5B (supplementary data) present the results of the calculation of the thermodynamic parameter values. The endothermic character of the Pb^2+^ adsorption mechanism is implied by the positive value of ΔHº. However, during adsorption, the sorbent's internal structure becomes more random, which results in ion replacement (Sachit 2024). This is confirmed by the positive values for (ΔSº). Addionally, the ΔHº value is more than 40 kJ/mol. This implied that chemisorption adsorption is preferred in Pb^2+^ adsorption (Elias et al. 2023). Negative Gibbs free energy (ΔG^o^) values indicate a favorable and spontaneous adsorption of Pb^2+^. Furthermore, when temperature increases, ΔGº values decrease, indicating that adsorption is more effective. The results is in good agreement with those published in other sources (Abdulkareem et al. 2019; Elias et al. 2023).

#### Adsorption mechanism

**Fig. 4:**
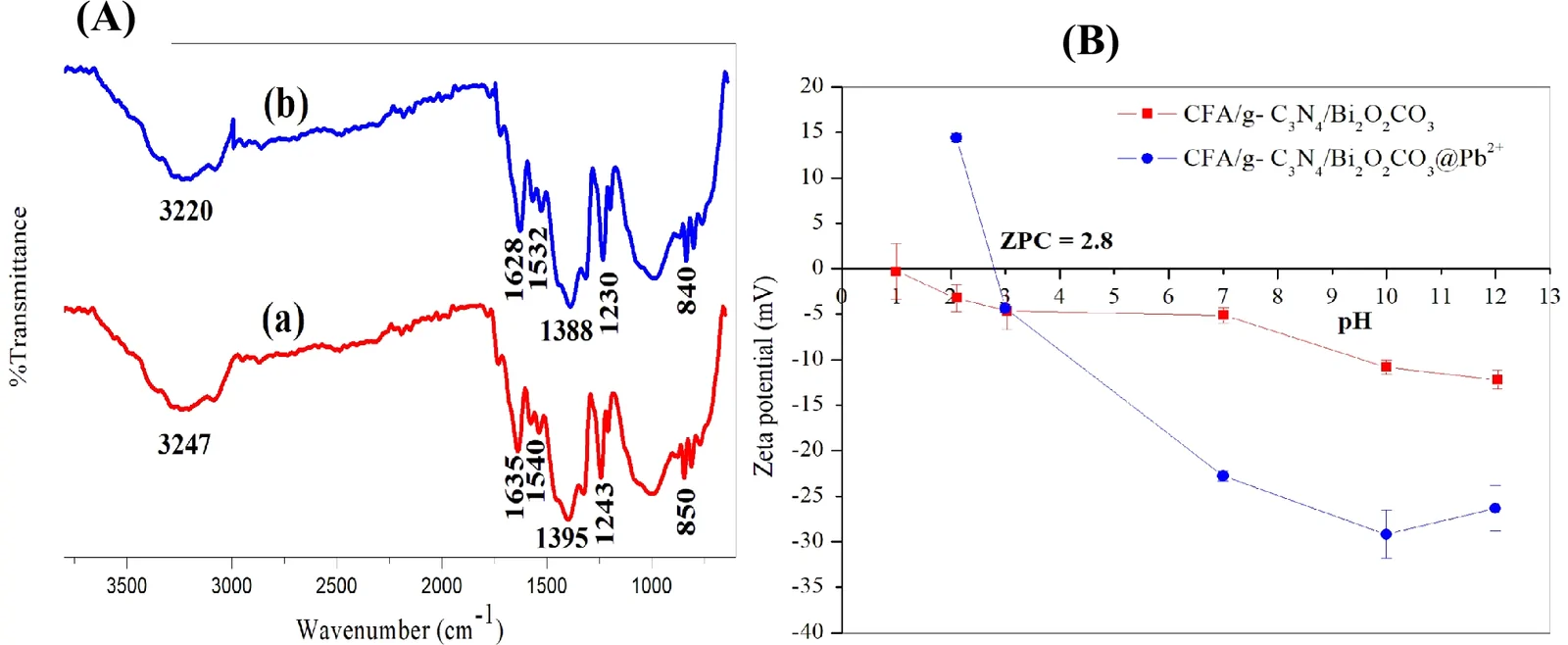
Characterization of CFA/g-C_3_N_4_/Bi_2_O_2_CO_3_ composite before and after Pb^2+^adsorption (**A**) Infrared spectra and (**B**) Zeta potential

To understand the interaction between CFA/g-C_3_N_4_/Bi_2_O_2_CO_3_ composite adsorbent and Pb^2+^, the composite adsorbent was analyzed before and after adsorption by FTIR, zeta potential, XRD, SEM–EDX analysis, and high-resolution XPS. Figure 4A shows the infrared spectra of CFA/g-C_3_N_4_/Bi_2_O_2_CO_3_ composite adsorbent before and after the adsorption. Compared to the spectrum of the adsorbent before Pb^2+^ adsorption, the absorption bands of N–H (3247 cm^−1^), C = N (1635 cm^−1^), C-N (1540 cm^−1^) and C-N (1243 cm^−1^) were redshifted respectively to 3220 cm^−1^, 1628 cm^−1^, 1532 cm^−1^ and 1230 cm^−1^, after adsorption. Also, Al–O–Si (1395 cm^−1^) and CO_3_^2−^ (850 cm^−1^) were downshifted to 1388 cm^−1^and 840 cm^−1^, respectively in the FTIR spectrum of CFA/g-C_3_N_4_/Bi_2_O_2_CO_3_@Pb^2+^ composite. These redshift changes in –NH_2_, –C = N and –C-N (heterocycles units), Al–O–Si and CO_3_^2−^ groups indicate that the surface groups of the adsorbent provide lone pair for Pb^2+^ ion. Thus, the complexation between Pb^2+^ ions and the functional groups of the adsorbent is one of the driving forces for the adsorption of Pb^2+^ ions.

Figure 4B presents the variation of zeta potential at different pH values of the CFA/g-C_3_N_4_/Bi_2_O_2_CO_3_ composite adsorbent before and after Pb^2+^ adsorption. The results reveal that the surface of the composite adsorbent is negatively charged at all pH values, thus promoting the electrostatic attraction between the adsorben surface and Pb^2+^ ions. The negative charge on the composite adsorbent can be explained by the presence of functional groups (such as –OH, NH_2_, -C = N, and –CO_3_^2−^) on the surface of the material. After adsorption of Pb^2+^ ions, the electronegativity of the surface was decreased (pH_ZPC_ = 2.8). This indicates that Pb^2+^ ions are strongly attracted to the negative charges on the surface of CFA/g-C_3_N_4_/Bi_2_O_2_CO_3_.

**XRD** analysis of CFA/g-C_3_N_4_/Bi_2_O_2_CO_3_ composite before and after adsorption was compared to elucidate the adsorption mechanism (Fig. 1A(c–d)). It was found that the XRD peak intensities decreased after adsorption. This is because the attached Pb^2+^ ion interacts with the surface of the composite adsorbent, resulting in an insignificant alteration in the XRD spectrum. The elemental composition of CFA/g-C_3_N_4_/Bi_2_O_2_CO_3_@Pb^2+^ composite was conducted using EDX (Fig. 2f). The results reveal that the CFA/g-C_3_N_4_/Bi_2_O_2_CO_3_@Pb^2+^ composite contains 14.3% lead, thus verifying that the Pb^2+^ ion was successfully adsorbed to the CFA/g-C_3_N_4_/Bi_2_O_2_CO_3_ surface.

**Fig. 5:**
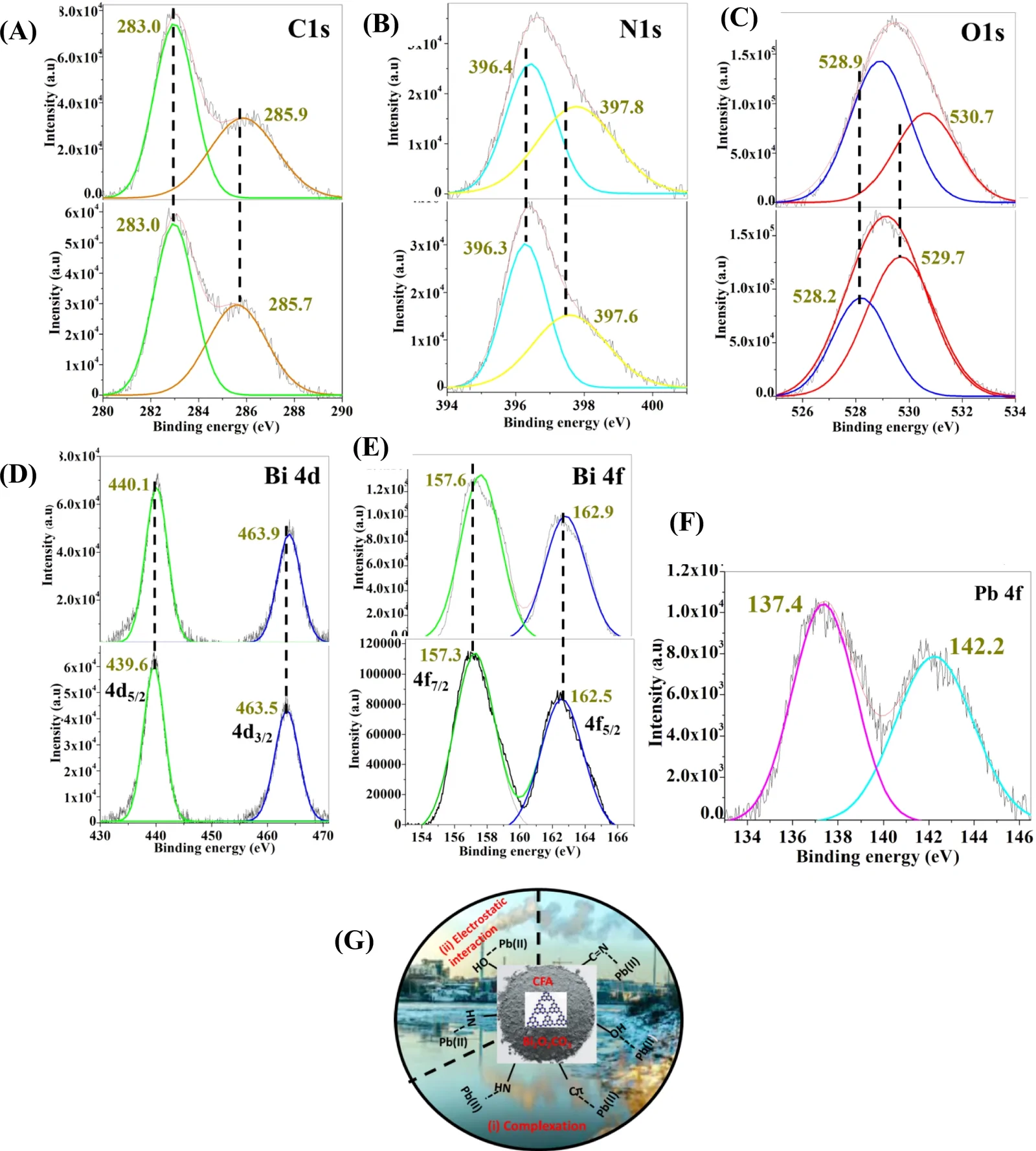
(**A**–**G**). XPS spectra of CFA/g-C_3_N_4_/Bi_2_O_2_CO_3_ composite before and after Pb^2+^ adsorption fitting curves of (**A**) C 1 s, (**B**) N 1 s, (**C**) O 1 s, (**D**) Bi 4 d, (**E**) Bi 4f, and (**F**) Pb 4f. (**G**) Possible adsorption mechanism

**XPS** analysis was further used to understand the adsorption mechanism of Pb^2+^ on the as-synthesized composite material. In the deconvoluted XPS spectrum C 1S (Fig. 5A), two peaks are noticed at 283.0 eV (C–C, C-N, C-O) and 285.7 eV (C = N, C = O). After metal adsorption, the peak at 285.7 eV migrates to 285.9 eV because of the complexation the Pb^2+^ ions to the nitrogen atoms that form a bond with the carbon. The O1s spectrum before and after adsorption of Pb^2+^ is displayed in Fig. 5C. The peaks before adsorption showed peaks at 528.2 eV (C––O) and 529.7 eV (C = O), while the peaks after adsorption upward shift to 528.9 eV and 530.7 eV 530.60 eV, indicating the co-ordination Pb^2+^ ion to the oxygen-containing functional groups. The deconvolution of N 1 s spectrum (Fig. 5D) showed peaks at 396.3 and 397.6 indexing to the N–C and N = C bonds moved to 396.4 eV and 397.9 eV respectively after adsorbing Pb^2+^. This is also due to the chelation of nitrogen- containing functional groups in CFA/g-C_3_N_4_/Bi_2_O_2_CO_3_ composite to Pb^2+^ ions. The high-resolution Bi_4d_ and Bi_4f_ spectra before and after Pb^2+^ adsorption are resolved into four peaks (Fig. 5D and 5E); the peaks at approximately 439.9 eV and 463.5 eV which belong to Bi4d_5/2_ and Bi4d_3/2_, respectively moves to 440.1 and 463.9 after adsorption (shown in Fig. 5D). In addition, the peaks located 157.3 eV and 162.5 eV corresponding to Bi4f_7/2_ and Bi4f_5/2_ respectively are shifted to slightly higher binding energy of 157.6 eV and 162.9 eV, after adsorption (Fig. 5E). This indicates that the Pb^2+^ ion is be anchored to the surface by the carbonate in the Bi_2_O_2_CO_3_ to form Pb- Bi_2_O_2_CO_3_-C_3_N_4_/CFA bonds. The spectrum of Pb 4f is presented in Fig. 5G, and the main peaks appear at 137.4 eV and 142.2 eV after adsorbing Pb^2+^confirms that Pb^2+^ is adsorbed on CFA/g-C_3_N_4_/Bi_2_O_2_CO_3_ composite. In conclusion, the electron-rich N and O-containing functional groups in CFA/g-C_3_N_4_/Bi_2_O_2_CO_3_ composite play a vital role in the uptake of Pb^2+^ metal ions. Based on the above analysis, we conclude that two possible interaction mechanisms (Fig. 5G) govern the adsorption of Pb^2+^ ions on the surface of CFA/g-C_3_N_4_/Bi_2_O_2_CO_3_, which are surface complexation and electrostatic attraction.

### Reusability of of CFA/g-C_3_N_4_/Bi_2_O_2_CO_3_@Pb^2+^ composite for photocatalytic degradation of RhB and latent fingerprint detection

#### Verification of charge transfer and separation

##### UV–Visible diffuse reflectance (DRS) study

**Fig. 6:**
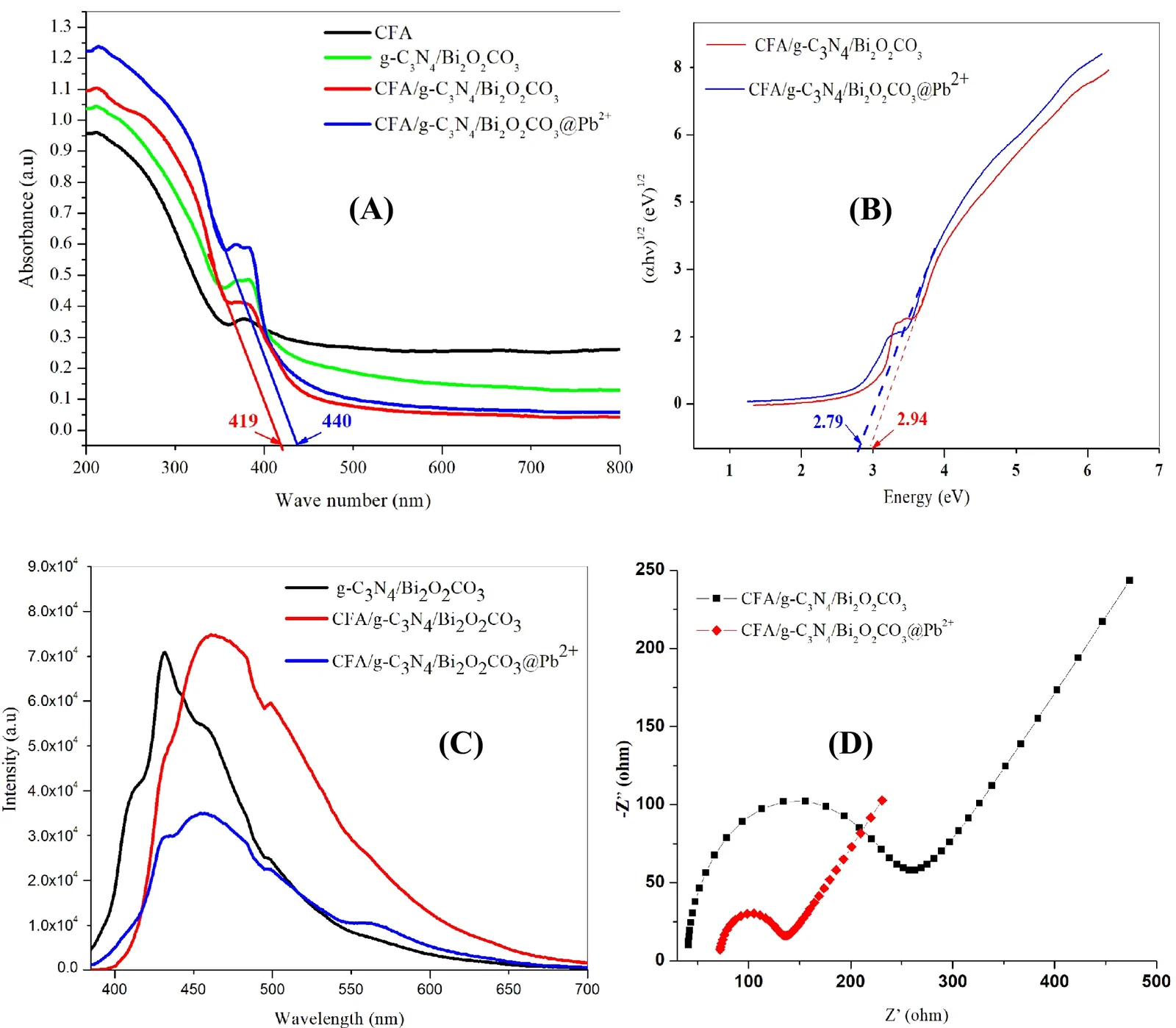
(**A**–**C**), (**A**) UV–vis diffuse reflectance spectra of CFA, g-C_3_N_4_/Bi_2_O_2_CO_3_, CFA/g-C_3_N_4_/Bi_2_O_2_CO_3_ and CFA/g-C_3_N_4_/Bi_2_O_2_CO_3_@Pb^2+^*.*(**B**) Tauc’s plot of CFA/g-C_3_N_4_/Bi_2_O_2_CO_3_ and CFA/g-C_3_N_4_/Bi_2_O_2_CO_3_@Pb^2+^*.* (**C**) fluorescence spectra of g-C_3_N_4_/Bi_2_O_2_CO_3_, CFA/g-C_3_N_4_/Bi_2_O_2_CO_3_ and CFA/g-C_3_N_4_/Bi_2_O_2_CO_3_@Pb^2+^and (**D**) The EIS Nyquist plot of CFA/g-C_3_N_4_/Bi_2_O_2_CO_3_ and CFA/g-C_3_N_4_/Bi_2_O_2_CO_3_@Pb^2+^ in KCl

The optoelectronic studies of the CFA, g-C_3_N_4_/Bi_2_O_2_CO_3_, CFA/g-C_3_N_4_/Bi_2_O_2_CO_3_ and the CFA/g-C_3_N_4_/Bi_2_O_2_CO_3_@Pb^2+^ composites were carried out using UV–vis DRS (Fig. 6A). Pure CFA shows a typical absorption peak at 390 nm, while g-C_3_N_4_/Bi_2_O_2_CO_3_ has absorption edge at 416 nm. The optical absorption edge of the CFA/g-C_3_N_4_/Bi_2_O_2_CO_3_ composite shows a systematic slight red-shift relative to the g-C_3_N_4_/Bi_2_O_2_CO_3_ and absorbs UV light at 419 nm. This could be due to the introduction of CFA onto the g-C_3_N_4_/Bi_2_O_2_CO_3_ composite. Compared with that of CFA/g-C_3_N_4_/Bi_2_O_2_CO_3_, the absorption edge of CFA/g-C_3_N_4_/Bi_2_O_2_CO_3_@Pb^2+^ is redshifted to 440 nm. The improved absorption edge of the CFA/g-C_3_N_4_/Bi_2_O_2_CO_3_@Pb^2+^ photocatalysts indicates that Pb^2+^ can serve as an effective visible-light sensibilize, thus improving the photoelectrochemical properties of the composites.

The band gap energy (E_g_) is a measure of the optical performance of a semiconductor. The E_g_ is estimated via Tauc’s plot shown in Fig. 6B, derived given by formula (9) below (Babu et al. 2018):

9 $$\alpha hv = \mathrm{A}{(h \upnu - {\mathrm{E}}_{\mathrm{g}})}^{1/\mathrm{n}}$$

where α is the absorption coefficient, 1/n is ½ is indirect allowed transition, h is Plank constant, ν is light frequency and A is a constant function.

The CFA/g-C_3_N_4_/Bi_2_O_2_CO_3_ and CFA/g-C_3_N_4_/Bi_2_O_2_CO_3_@Pb^2+^ photocatalysts exhibited a low band gap energy of 2.94 eV and 2.79 eV, respectively, due to a semi-conductor like behavior of these photocatalysts. The obtained E_g_ value of CFA/g-C_3_N_4_/Bi_2_O_2_CO_3_@Pb^2+^ is comparable to g-C_3_N_4/_TiO_2_ composite photocatalysts previously reported in the literature (Giannakopoulou et al. 2017). It is also evidence that the presence of a minute amount of Pb^2+^ on the CFA/g-C_3_N_4_/Bi_2_O_2_CO_3_ composite surface reduces the band gap. This phenomenon could participate in decreasing the electrons-holes recombination rate and enhance the photocatalytic activity using low-energy irradiation sources (Prabakaran et al. 2021).

##### Photoluminescence

PL was used to evaluate charge recombination rates of the electron-holes in the as-prepared g-C_3_N_4_/Bi_2_O_2_CO_3_, CFA/g-C_3_N_4_/Bi_2_O_2_CO_3_ and CFA/g-C_3_N_4_/Bi_2_O_2_CO_3_@Pb^2+^ composites (Fig. 6C). The analyses were performed in ethanol at an excitation wavelength of 360 nm. Generally, the lower PL intensity means low charge carrier recombinations, higher photogenerated carriers’ efficiency and a higher charge separation (Iqbal et al. 2019; Liu et al. 2019). In relation to the g-C_3_N_4_/Bi_2_O_2_CO_3_, CFA/g-C_3_N_4_/Bi_2_O_2_CO_3_ heterostructure composites shows low PL intensity, which signifies slower recombination rates of photo-induced charge carriers. The observed decrease in PL can be related to the reduction in the concentration of g-C_3_N_4_/Bi_2_O_2_CO_3_ in the composite upon dispersion CFA loading onto g-C_3_N_4_/Bi_2_O_2_CO_3_ composite. It is also noted that the recombination rate for CFA/g-C_3_N_4_/Bi_2_O_2_CO_3_@Pb^2+^ composite was quenched ~ 2 times compared to the composite before adsorption. This could primarily be accredited to the Pb^2+^ metal ion which hinders the recombination of electron-holes pairs in the composite. This feature shows the efficiency of the CFA/g-C_3_N_4_/Bi_2_O_2_CO_3_@Pb^2+^ composite in the photocatalytic degradation of organic substances (Umejuru et al. 2021). On the other hand, the higher PL intensity of CFA/g-C_3_N_4_/Bi_2_O_2_CO_3_ composite signifies that the pristine adsorbent has an unfavourable recombination rate of photoinduced electron–hole pairs.

##### Electrochemical impedance spectroscopy (EIS)

EIS was applied to assess the charge transfer activities of the as-prepared photocatalyst. In Fig. 6D, the charge transfer resistance for CFA/g-C_3_N_4_/Bi_2_O_2_CO_3_@Pb^2+^ and CFA/g-C_3_N_4_/Bi_2_O_2_CO_3_ composites are 100 Ω and 150 Ω respectively. In addition, the CFA/g-C_3_N_4_/Bi_2_O_2_CO_3_@Pb^2+^ composite shows a considerably smaller semicircle diameter than that of CFA/g-C_3_N_4_/Bi_2_O_2_CO_3_, signifying smaller charge-transfer resistance (R_ct_) compared to the pristine as-prepared composite (Makola et al. 2022). The improved conductivity of the CFA/g-C_3_N_4_/Bi_2_O_2_CO_3_@Pb^2+^ composite is possibly due to the Pb^2+^ ions on the surface of the CFA/g-C_3_N_4_/Bi_2_O_2_CO_3_ composite. Overall, the CFA/g-C_3_N_4_/Bi_2_O_2_CO_3_@Pb^2+^ composite demonstrated useful boundary charge transfer compared to the pristine adsorbent. Furthermore, the CFA/g-C_3_N_4_/Bi_2_O_2_CO_3_@Pb^2+^ photocatalyst exhibits an improved electron–hole pair separation (Oh et al. 2019; Wang et al. 2017).

#### Photocatalytic degradation of RhB

##### Photocatalytic performance for RhB degradation

**Fig. 7:**
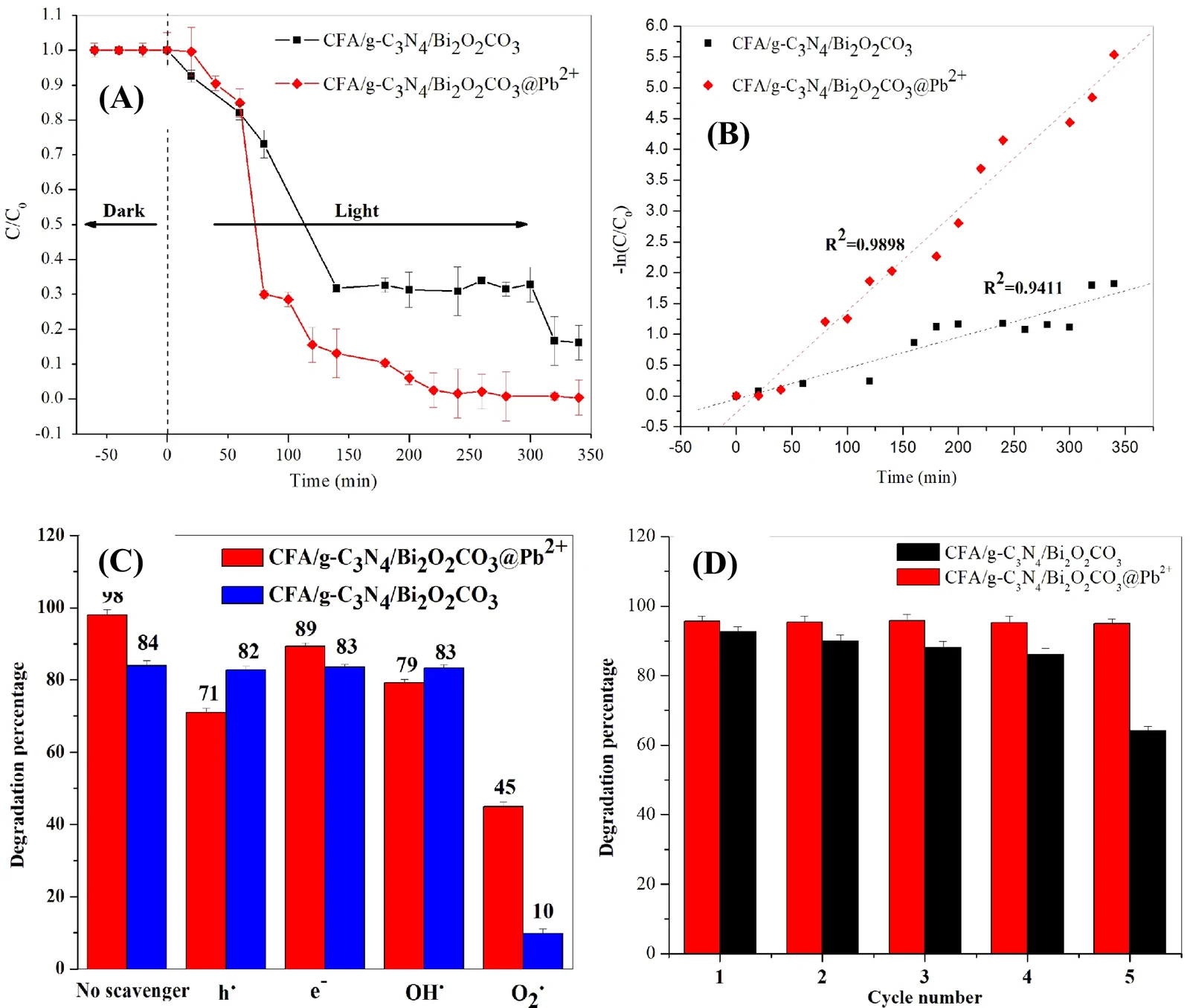
(**A**–**D**), (**A**) Degradation efficiency and (**B**) kinetics plots of decomposition of RhB by CFA/g-C_3_N_4_/Bi_2_O_2_CO_3_ and CFA/g-C_3_N_4_/Bi_2_O_2_CO_3_@Pb^2+^ photocatalysts, (**C**) effect of scavengers radical for RhB degradation over CFA/g-C_3_N_4_/Bi_2_O_2_CO_3_@Pb^2+^ and (**D**) reusability of CFA/g-C_3_N_4_/Bi_2_O_2_CO_3_ and CFA/g-C_3_N_4_/Bi_2_O_2_CO_3_@Pb^2+^ for the photocatalytic degradation of RhB through five cycles. Reaction conditions: T = 20 °C, dosage 0.05 g, RhB concentration = 5 mg L^−1^ under visible light

The degradation ability of the CFA/g-C_3_N_4_/Bi_2_O_2_CO_3_@Pb^2+^ and CFA/g-C_3_N_4_/Bi_2_O_2_CO_3_ was examined in RhB removal. Figure 7A depicts the degradation rates plot of RhB over the CFA/g-C_3_N_4_/Bi_2_O_2_CO_3_ and CFA/g-C_3_N_4_/Bi_2_O_2_CO_3_@Pb^2+^ composites. As seen, the CFA/g-C_3_N_4_/Bi_2_O_2_CO_3_@Pb^2+^ displays superior photocatalytic ability than the pristine CFA/g-C_3_N_4_/Bi_2_O_2_CO_3_ in the degradation of RhB. This suggests that the loaded amount of Pb on the spent composite plays a key role in boosting the photocatalytic performance of the composite. It is also evident that the degradation rate was fast during the first 80 min after visible light irradiations; and the removal of RhB over CFA/g-C_3_N_4_/Bi_2_O_2_CO_3_@Pb^2+^ and CFA/g-C_3_N_4_/Bi_2_O_2_CO_3_ were 70% and 20%, respectively. Then it declined gradually, and it remained constant after 240 min of light irradiation.

The degradation of organic pollutants usually agrees with the pseudo-first-order-kinetics equation, which can be described as (Shoja et al. 2019):

10 $$-\mathrm{I}\mathrm{n}(\mathrm{C} /{\mathrm{C}}_{0})\hspace{0.17em}=\hspace{0.17em}\mathrm{k}\mathrm{t}$$

where t is the degradation time and k denotes to the first-order-kinetics reaction rate constant. As seen from Fig. 7B, the plot of -In(C/C_0_) vs. t is linear with high coefficient of determination *R*^2^˃ 0.94. This indicates the degradation of RhB is best described by the pseudo first order equation. The rate constant of CFA/g-C_3_N_4_/Bi_2_O_2_CO_3_@Pb^2+^ composite (0.0165 min^−1^) is higher, that is almost 3.3-folds greater than that of the pristine CFA/g-C_3_N_4_/Bi_2_O_2_CO_3_ (0.0050 min^−1^). Kinetic results confirmed that the presence of Pb^2+^ ions on the spent adsorbent could significantly enhance the photocatalytic performance of pristine CFA/g-C_3_N_4_/Bi_2_O_2_CO_3_ under visible light irritation. According to DRS analysis, the improvement in photocatalytic performance of CFA/g-C_3_N_4_/Bi_2_O_2_CO_3_@Pb^2+^ composite may be attributed to its narrower band gap energy than that of pristine CFA/g-C_3_N_4_/Bi_2_O_2_CO_3_ composite, enabling the electrons and holes generation. Moreover, PL and EIS analysis revealed the low recombination rate in CFA/g-C_3_N_4_/Bi_2_O_2_CO_3_@Pb^2+^ composite and improved charge separation, thus boosting the photocatalytic performance.

##### Trapping experiment

The photocatalytic degradation of RhB dye depends on the existence of a radical active species produced by the as-synthesized composite. A radical scavenger experiment was performed to explore the role of active species in the RhB degradation process by CFA/g-C_3_N_4_/Bi_2_O_2_CO_3 _and CFA/g-C_3_N_4_/Bi_2_O_2_CO_3_@Pb^2+^ composites. This experiment was conducted with the addition of AgNO_3_, ethylene diamine tetra acetic acid (EDTA), isopropanol alcohol (IPA) and L-Ascorbic acid were applied to quench radicals e^−^, h^+^, ^•^OH and ^•^${\mathrm{O}}_{2}^{-}$, respectively (Sangani et al. 2024; Zou et al. 2023). The removal efficiency in the presence of the trapping radicals was determined and the obtained results are presented in Fig. 7C. The results revealed that the degradation efficiency of RhB over CFA/g-C_3_N_4_/Bi_2_O_2_CO_3_@Pb^2+^ composite decreased in the following order: L-Ascorbic (45%) > EDTA (71%) > IPA (79%) > AgNO_3_ (89%) compared with no scavengers (98%). This indicated that superoxide free radicals •${\mathrm{O}}_{2}^{-}$ play a vital contribution to the degradation process. Further, the h^+^ and OH^•^ reactive species also suppress the degradation process, whereas the effect of the generated e^−^ on this process is relatively negligible. Thus, RhB degradation was triggered by the formation of •O_2_^−^, ^•^OH and h^+^ reactive radical species (Eqs. (11)–(13)).

However, the degradation efficiency of RhB over pristine CFA/g-C_3_N_4_/Bi_2_O_2_CO_3_@Pb^2+^ composite is significantly decreased in the presence of L-Ascorbic acid (10%), while the introduction of the other scavengers has no effect in suppressing the RhB degradation. This means only, however, the degradation efficiency of RhB over pristine CFA/g-C_3_N_4_/Bi_2_O_2_CO_3_@Pb^2+^ composite is significantly decreased in the presence of L-Ascorbic acid (10%), while the introduction of the other scavengers has minimal impact in suppressing the RhB degradation. This means only •${\mathrm{O}}_{2}^{-}$ species contributes to the photocatalytic process. These findings demonstrate that lead on the loaded spent composite encourages the production of the photogenerated •O_2_^−^, •OH and h +, thus boosting the photocatalytic activity.

Overall, the CFA/g-C_3_N_4_/Bi_2_O_2_CO_3_@Pb^2+^ photocatalyst absorbs visible light triggering the generation of negative-electrons (e^−^) and positive-holes (h^+^) pairs. The photogenerated electrons and holes located on the valence band (CB) and conduction band (VB), respectively attack oxyl radicals (OH•), superoxide radicals (•O_2_^−^) and hydroperoxyl radicals (•OOH) (Liang et al. 2018). The RhB can be oxidized by the photo-generated ROS. Based on the quenching test, the plausible photocatalytic degradation of RhB over CFA/g-C_3_N_4_/Bi_2_O_2_CO_3_@Pb^2+^ can be summarized as Eqs. ((11)–(18)) (Chen et al. 2024; Liang et al. 2018; Sre et al. 2024).

11 $$\mathrm{C}\mathrm{F}\mathrm{A}/\mathrm{g}-{\mathrm{C}}_{3}{\mathrm{N}}_{4}/{\mathrm{B}\mathrm{i}}_{2}{\mathrm{O}}_{2}{\mathrm{CO}}_{3}@{\mathrm{P}\mathrm{b}}^{2+}\hspace{0.17em}+\hspace{0.17em}\mathrm{h}\upnu \hspace{0.17em}\to \hspace{0.17em}\mathrm{C}\mathrm{F}\mathrm{A}/\mathrm{g}-{\mathrm{C}}_{3}{\mathrm{N}}_{4}/{\mathrm{B}\mathrm{i}}_{2}{\mathrm{O}}_{2}{\mathrm{C}\mathrm{O}}_{3}@{\mathrm{P}\mathrm{b}}^{2+}{({\mathrm{h}}^{+})}_{\mathrm{V}\mathrm{B}}+{\mathrm{e}}_{\mathrm{C}\mathrm{B}}^{-}$$

12 $$({{\mathrm{O}}_{2})}_{\mathrm{a}\mathrm{q}} + {\mathrm{e}}_{\mathrm{C}\mathrm{B}}^{-} \hspace{0.17em}\to \hspace{0.17em}\bullet {{\mathrm{O}}_{2}}^{-}$$

13 $$\mathrm{C}\mathrm{F}\mathrm{A}/\mathrm{g}-{\mathrm{C}}_{3}{\mathrm{N}}_{4}/{\mathrm{B}\mathrm{i}}_{2}{\mathrm{O}}_{2}{\mathrm{C}\mathrm{O}}_{3}@{\mathrm{P}\mathrm{b}}^{2+}{({\mathrm{h}}^{+})}_{\mathrm{V}\mathrm{B}}\hspace{0.17em}+\hspace{0.17em}{\mathrm{H}}_{2}\mathrm{O}\hspace{0.17em}\to \hspace{0.17em}\mathrm{O}\mathrm{H}\bullet \hspace{0.17em}+\hspace{0.17em}{\mathrm{H}}^{+}$$

14 $$\mathrm{C}\mathrm{F}\mathrm{A}/\mathrm{g}-{\mathrm{C}}_{3}{\mathrm{N}}_{4}/{\mathrm{B}\mathrm{i}}_{2}{\mathrm{O}}_{2}{\mathrm{C}\mathrm{O}}_{3}@{\mathrm{P}\mathrm{b}}^{2+}{({\mathrm{h}}^{+})}_{\mathrm{V}\mathrm{B}\hspace{0.17em}}+\hspace{0.17em}{\mathrm{O}\mathrm{H}}^{-\hspace{0.17em}}\leftrightarrow \hspace{0.17em}{\mathrm{O}\mathrm{H}}^{\bullet }$$

15 $$\bullet {{\mathrm{O}}_{2}}^{-}\hspace{0.17em}+\hspace{0.17em}{\mathrm{H}}^{+} \hspace{0.17em}\to \hspace{0.17em}\bullet \mathrm{O}\mathrm{O}\mathrm{H}$$

16 $$\bullet \mathrm{O}\mathrm{O}\mathrm{H}\hspace{0.17em}{+}^{\bullet }\hspace{0.17em}{{\mathrm{O}}_{2}}^{-}\hspace{0.17em}+\hspace{0.17em}{\mathrm{H}}^{+} \hspace{0.17em}\to \hspace{0.17em}{\mathrm{O}}_{2}\hspace{0.17em}+\hspace{0.17em}{\mathrm{H}}_{2}{\mathrm{O}}_{2}$$

17 $${\mathrm{H}}_{2}{\mathrm{O}}_{2}\hspace{0.17em}+\hspace{0.17em}\bullet {{\mathrm{O}}_{2}}^{-}\hspace{0.17em}\to \hspace{0.17em}\mathrm{O}\mathrm{H}\bullet \hspace{0.17em}+\hspace{0.17em}{\mathrm{O}\mathrm{H}}^{-}\hspace{0.17em}+\hspace{0.17em}{\mathrm{O}}_{2}$$

18 $$\bullet {{\mathrm{O}}_{2}}^{-}\hspace{0.17em}+\hspace{0.17em}\mathrm{O}\mathrm{H}\bullet \hspace{0.17em}+\hspace{0.17em}{\mathrm{h}}^{+}\hspace{0.17em}+\hspace{0.17em}\mathrm{R}\mathrm{h}\mathrm{B}\hspace{0.17em}\to \hspace{0.17em}\mathrm{d}\mathrm{e}\mathrm{g}\mathrm{r}\mathrm{a}\mathrm{d}\mathrm{e}\mathrm{d} \mathrm{p}\mathrm{r}\mathrm{o}\mathrm{d}\mathrm{u}\mathrm{c}\mathrm{t}\mathrm{s}\hspace{0.17em}+\hspace{0.17em}{\mathrm{H}}_{2}\mathrm{O}$$

##### Recyclability

To investigate the durability and reusability of the CFA/g-C_3_N_4_/Bi_2_O_2_CO_3_ and CFA/g-C_3_N_4_/Bi_2_O_2_CO_3_@Pb^2+^ composites, the trend of degradation RhB under similar reaction conditions was examined, and the results are presented in Fig. 7D. As seen, the photocatalytic performance of the CFA/g-C_3_N_4_/Bi_2_O_2_CO_3_@Pb^2+^ composite in degrading RhB is slightly decreased after 5 re-use cycles, demonstrating high durability of the lead loaded spent composite. However, the pristine CFA/g-C_3_N_4_/Bi_2_O_2_CO_3_ composite displayed a significant decline in photocatalytic degradation of RhB (approximately 65.0%) in comparison to the first degradation cycle. Therefore, the CFA/g-C_3_N_4_/Bi_2_O_2_CO_3_@Pb^2+^ composite has noteworthy potential usages in future.

To evaluate photocorrosion of the composite photocatalyst during catalytic operation, the concentration of Pb^2^⁺ in the aqueous phase was measured by ICP-OES after each reaction cycle. No detectable leaching of Pb^2^⁺ (< 0.01 mg L⁻^1^) into the solution was observed after five consecutive cycles. The high photostability of CFA/g-C_3_N_4_/Bi_2_O_2_CO_3_@Pb^2+^ may be attributed to the synergistic effect of the nitrogen-rich g-C₃N₄ component, which enhances the preferential adsorption of Pb^2^⁺ on the catalyst surface. This strong adsorption may reduce photocorrosive degradation by limiting the release of Pb^2^⁺ from the composite.

##### Optimization photocatalytic degradation of RhB using CCD

Table 2 (supplementary data) displays the ANOVA result obtained from the CCD experimental design for RhB photodegradation over CFA/g-C_3_N_4_/Bi_2_O_2_CO_3_@Pb^2+^. As seen, the model terms, including Exposure time (B), Exposure time*Dosage of catalyst (AB), (Exposure time)^2^ (A)^2^, (Dosage of catalyst)^2^ (B)^2^, (pH)^2^ (C)^2^ and Dosage of catalyst*(Exposure time)^2^ (AB^2^) have p-values less than 0.05; indicating that they are significant independent variables affected for RhB dye degradation efficiency, with p-values less than 0.05. As a result, the relationship between the test variables and RhB dye degradation efficiency (response) was expressed by the second-order polynomial model equation as follows Eq. (19):

**Table 2:** Analysis of variance ANOVA for Response Surface Reduced Cubic Model (Aliased)

Source	Sum of Squares	Mean df	*F* Square	*p*-value Value	Prob > F
Model	6228.35	10	622.84	59.50	< 0.0001
A-Exposure time	3872.00	1	3872.00	369.92	< 0.0001
B-Dosage of catalysis	2.17	1	2.17	0.21	0.6594
C-pH	6.92	1	6.92	0.66	0.4370
AB 56.18	1	56.18	5.37	0.0457	
AC 1.28	1	1.28	0.12	0.7346	
BC 11.05	1	11.05	1.06	0.3311	
A^2^ 1639.43	1	1639.43	156.63	< 0.0001	
B^2^ 119.73	1	119.73	11.44	0.0081	
C^2^ 240.41	1	240.41	22.97	0.0010	
AB^2^ 2837.52	1	2837.52	271.09	< 0.0001	
Residual	94.20	9	10.47		
Lack of Fit	67.25	4	16.81	3.12	0.1220
Pure Error	26.96	5	5.39		
Cor Total	6322.55	19			

*R*^2 =^ 0.9851 *R*^2^ Adj = 0.9685 C.V % = 3.76 Adeq Precision = 36.677

19 $$\mathrm{D}\mathrm{e}\mathrm{g}\mathrm{r}\mathrm{a}\mathrm{d}\mathrm{a}\mathrm{t}\mathrm{i}\mathrm{o}\mathrm{n} \mathrm{e}\mathrm{f}\mathrm{f}\mathrm{i}\mathrm{c}\mathrm{i}\mathrm{e}\mathrm{n}\mathrm{c}\mathrm{y} \mathrm{o}\mathrm{f} \mathrm{R}\mathrm{h}\mathrm{B} \left(\mathrm{\%}\right)\hspace{0.17em}=\hspace{0.17em}\hspace{0.17em}+\hspace{0.17em}88.60\hspace{0.17em}+\hspace{0.17em}26.16\mathrm{A}-0.40\mathrm{B}-0.71\mathrm{C}-2.65\mathrm{A}\mathrm{B}-0.40\mathrm{A}\mathrm{C}-1.17\mathrm{B}\mathrm{C}-10.67{\mathrm{A}}^{2}\hspace{0.17em}+\hspace{0.17em}2.{88\mathrm{B}}^{2}\hspace{0.17em}+\hspace{0.17em}4.08{\mathrm{C}}^{2}-29.26{\mathrm{AB}}^{2}$$

The effectiveness and reliability of the model were justified by residue analysis, allowing for the calculation of the coefficient of determination (*R*^2^). The model’s coefficient of determination (*R*^2^) is extremely high (0.9851), indicating that this cubic regression model accounts for 98.51% of the total variation. The adjusted coefficient (Adj *R*^2^) of 0.9685 indicates strong agreement between predicted and experimental values. Besides, the model is regarded as statistically significant as its Probability (*p*-value) is less than 0.05 at 95% confidence (Al-Ahmed et al. 2024; Xiao et al. 2017). The LOF for *p*-value was greater than 0.05 (0.1220), making it statistically insignificant. Furthermore, the good values of CV% (3.76%) and Adequate Precision (36.677) indicate a good signal-to-noise ratio, confirming the model’s capability for predicting the variable within the chosen experiment range.

Fig. S6A depicts the distribution of predicted versus experimental values for the responses corresponding to RhB degradation efficiency. The points are spread essentially randomly along the line, and the cubic model gives a high correlation between experimental and predicted values. To validate the normality premise, the residuals were transformed into a normal probability plot (Fig. S6B). The residual plot provides a straight line, and corroborates the assumption of normalcy (Al-Ahmed et al. 2024).

**Fig. 8:**
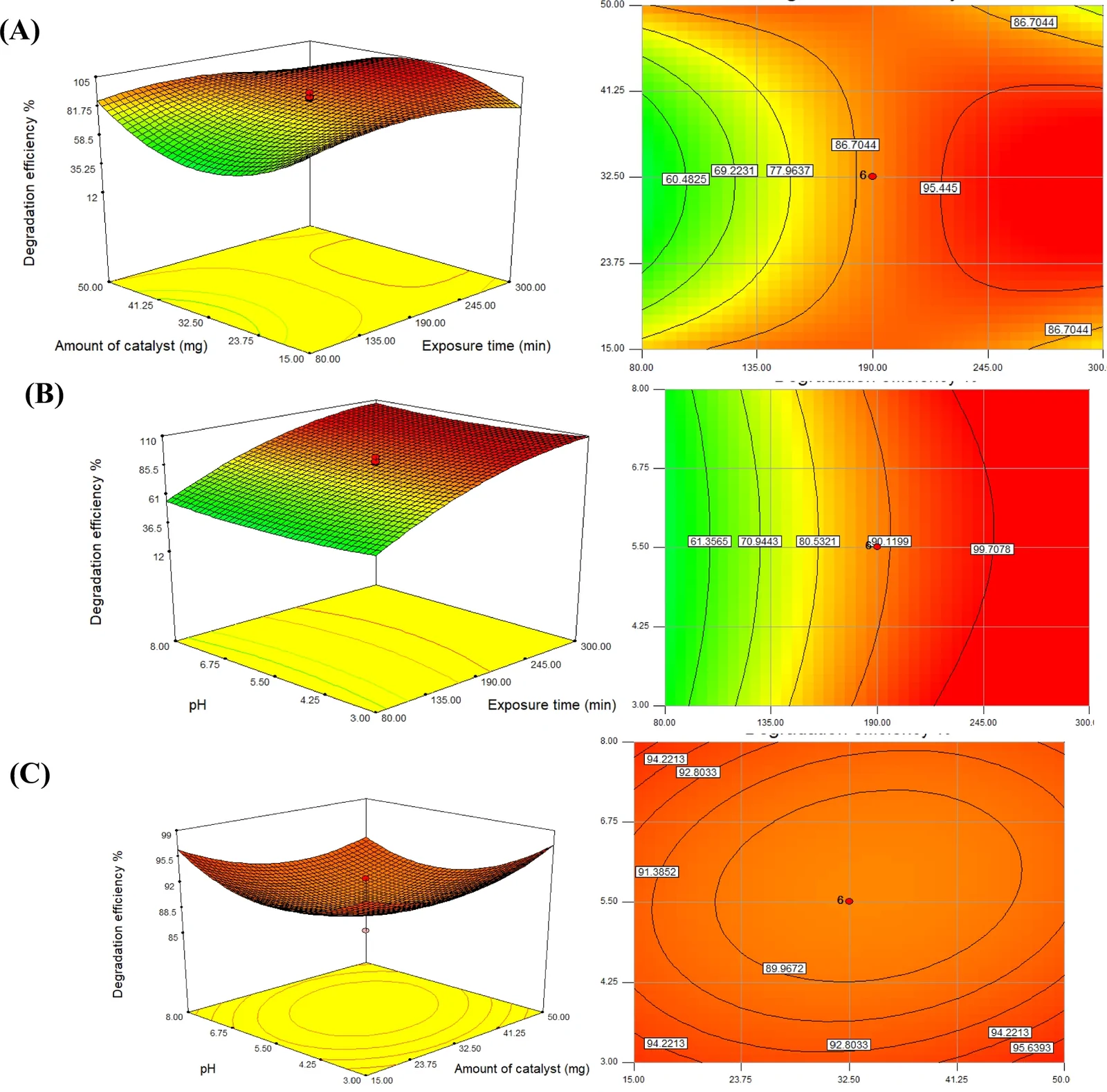
Surface plots (left panel) and contour plots (right panel) as a function of (**A**) amount of catalyst vs exposure time, (**B**) Exposure time vs initial pH, and (**C**) Initial pH vs amount of catalyst in photodegradation of RhB using CFA/g-C_3_N_4_/Bi_2_O_2_CO_3_@Pb^2^

Response surfaces are 3D or contour plots that display the change of a response with two factors while keeping the other factor constant. Figure 8 shows both 3D surface response and contours plots for RhB degradation efficiency (%) as a function of (A) amount of CFA/g-C_3_N_4_/Bi_2_O_2_CO_3_@Pb^2^ vs exposure time, (B) Exposure time vs initial pH, and (C) Initial pH vs amount of CFA/g-C_3_N_4_/Bi_2_O_2_CO_3_@Pb^2+^, respectively. As shown in Fig. 8A, the longer the irradiation time the higher the degradation efficiency (%) as a dye has sufficient time to occupy the active sites. The RhB degradation efficacy increased with the CFA/g-C_3_N_4_/Bi_2_O_2_CO_3_@Pb^2+^ dosage rising from 15 mg to 32.5 mg. This enhancement in degradation efficacy is caused by increased surface area, thus increasing the interaction of RhB molecules with the catalyst. However, further increase in catalyst dosage (from 32.5 mg to 50 mg) causes accumulation and occupying of the reactive sites, thus decreasing the degrading efficacy.

Figure 8B presents the combined effects of exposure time and pH on the RhB degradation efficiency. Clearly, the pH variations have only a minimal impact on the RhB degradation efficiency; nevertheless, increasing the exposure period considerably promotes RhB degradation. This implies that the degradation process is not pH-dependent in the domain under study. Finally, the interaction between CFA/g-C_3_N_4_/Bi_2_O_2_CO_3_@Pb^2+^ dosage and pH, as illustrated in Fig. 8C shows that the dosage of the catalyst has a significant effect on RhB degradation, while pH does not play a major role in the photodegrading process within the studied range.

Table 3 reports the optimum condition for RhB degradation over CFA/g-C_3_N_4_/Bi_2_O_2_CO_3_@Pb^2+^. Based on the results, the optimum values of the variables to achieve the maximum RhB degradation efficiency from an aqueous solution of 5 mg L^−1^ were; exposure time 260.92 min; amount of CFA/g-C_3_N_4_/Bi_2_O_2_CO_3_@Pb^2+^ 24.91 mg and solution pH 3.04 or 7.96 at 25°C. Because the model has an error (C.V% = 3.76), the predicted RhB degradation percentage exceeds 100%, when the experimental value is 100%. The good correlation between these two values of results suggests that the strategy to optimize the degradation process by Derringer’s desirability function approach for the removal of RhB in this work is successful**.**

**Table 3:** Optimum level of factors and photo degradation efficiency of RhB using CFA/g-C_3_N_4_/Bi_2_O_2_CO_3_@Pb^2+^

Solutions Number	Exposure time (min)	Amount of catalysis (mg)	pH	Photodegradation effeciency (%) Actual Calculated		RSD
1	260.92	24.91	3.04	100.00	103.35	2.29
2	260.92	24.91	7.96	100.00	102.44	1.68

The CFA/C_3_N_4_/Bi_2_O_2_CO_3_@Pb^2+^ composite performance in degrading RhB under optimum experimental conditions was compared with other photocatalysts, as shown in Table S8 (supplementary data). The CFA/C_3_N_4_/Bi_2_O_2_CO_3_@Pb^2+^ composite demonstrated higher photocatalytic efficiency than other photocatalysts, making it the ideal choice for removing organic pollutants from industrial wastewaters.

##### Photocatalytic removal of RhB in real sample

The performance of CFA/g-C_3_N_4_/Bi_2_O_2_CO_3_@Pb^2+^ composite on photocatalytic degradation of RhB was examined in municipal sewage water (collected from Pretoria city, South Africa) spiked with 5 mg L^−1^ of RhB. It was found that the CFA/g-C_3_N_4_/Bi_2_O_2_CO_3_@Pb^2+^ composite had photocatalytic removal of RhB dye of up to 99.9% within 240 min under visible light irradiation. This demonstrates the feasibility of a lead-loaded spent photocatalyst detoxifying RhB dye in a polluted water sample.

### Reusability of CFA/g-C_3_N_4_/Bi_2_O_2_CO_3_@Pb^2^ composite on latent fingerprint detection

Reusing this spent adsorbent in latent fingerprint (LFP) was essential to preventing secondary contamination once Pb^2+^ was successfully adsorbed onto CFA/g-C_3_N_4_/Bi_2_O_2_CO_3_. Because of well-developed photographs and discernible ridge patterns of LFP, legal innovation research has been required to examine suspects (Prabakaran & Pillay 2020).

To achieve this, diverse surfaces such as aluminium foil, glass slide, iron disc, and white tile were used for latent fingerprint detection, as shown in Fig. S7 (supplementary data). When CFA/g-C_3_N_4_/Bi_2_O_2_CO_3_@Pb^2+^ composite powder was applied to the surfaces, distinct fingerprint patterns were seen, and aluminium sheet and glass slide showed improved ridges. The CFA/g-C_3_N_4_/Bi_2_O_2_CO_3_@Pb^2+^ powder also demonstrated excellent sensitivity and efficiency compared to pristine adsorbent for LFP identification. According to this LFP characterization, the CFA/g-C_3_N_4_/Bi_2_O_2_CO_3_@Pb^2+^ composite could be used for forensic authentication.

## Cost estimation

A preliminary cost evaluation (Table S9 in supplementary data) estimated the production cost of 1 g of the CFA/g-C3N4/Bi2O2CO3 composite was ZAR 47.13 (approximately US$ 2.9/g) at lab scale. Bismuth nitrate and ethanol were the principal cost contributors, representing 61.9% and 36.8% of the overall material cost, respectively. However, large-scale production is expected to reduce the manufacturing cost to below US$ 1.5 g⁻^1^ through bulk procurement of reagents and the recovery and reuse of solvents. The synthesis cost of CFA/g-C_3_N_4_/Bi_2_O_2_CO_3_ was compared with those of zeolite A from CFA (129.0) (Sukchit et al. 2025), MFA-CS (112.0) (Sopanrao & Sreedhar 2025), Dodecyl dimethyl betaine and CS modified semi-carbonized fibres (175.8) (Li et al. 2024), ZnO-Cs (156.1) (Nguyen et al. 2023) and mesoporous silica from CFA (102.0) (Tenza et al. 2026), using a common cost basis of ZAR/g. This indicates that CFA/g-C_3_N_4_/Bi_2_O_2_CO_3_ has strong potential as an economical and scalable material for wastewater treatment.

Despite its promising cost profile, the industrial application of CFA/g-C_3_N_4_/Bi_2_O_2_CO_3_ requires a comprehensive techno-economic analysis that accounts for capital and operating expenses, scale-up requirements, labor, waste management, and lifecycle impacts. Future studies should also include sensitivity analyses to identify major cost drivers and assess economic feasibility under different operational and market conditions, thereby establishing the material’s commercial potential for large-scale wastewater treatment.

## Conclusion

In this study, the CFA/g-C_3_N_4_/Bi_2_O_2_CO_3_ composite has been synthesized by the hydrothermal reaction technique and applied to remove Pb(II) from aqueous solutions. The composite exhibited excellent adsorption capacities for the removal of Pb(II) metal in solution, with a maximum adsorption capacity value of 60.29 mg g⁻^1^ at 25 °C. The adsorption data was well described by the pseudo-second-order kinetic model and Langmuir isotherm, suggesting the chemisorption and monolayer coverage reaction. The values of ΔH^o^ and ΔG^o^ parameters demonstrated the endothermic and spontaneous nature of an adsorption process. The adsorption mechanism was elucidated using FT-IR and XPS analysis, and both the electrostatic interaction and complexation reaction are responsible for the adsorption. The removal of Pb^2+^ ions using CFA/g-C_3_N_4_/Bi_2_O_2_CO_3_ from real wastewater samples was also assessed. The Pb^2+^ ion-loaded spent adsorbent (CFA/g-C_3_N_4_/Bi_2_O_2_CO_3_**@**Pb^2+^) revealed excellent reusability and stability as a photocatalyst for degrading RhB. This CFA/g-C_3_N_4_/Bi_2_O_2_CO_3_@Pb^2^⁺ had < 99% RhB degradation efficiency within 260 min and stability up to 5 cycles. Additionally, the CFA/g-C_3_N_4_/Bi_2_O_2_CO_3_**@**Pb^2+^ was utilized as a labeling agent for latent fingerprint detection under daylight. This now demonstrates a new route to reduce secondary pollution problems by reusing spent adsorbents for mitigation dyes in wastewaters and as an effective labeling agent for LFP detection in forensic investigations.

Further studies should evaluate Pb^2^⁺ adsorption in continuous-flow column systems to assess the practical performance of CFA/g-C_3_N_4_/Bi_2_O_2_CO_3_ composite material, with particular emphasis on techno-economic feasibility and long-term sustainability. In addition, the low charge-transfer resistance observed by electrochemical impedance spectroscopy suggests that the CFA/g-C_3_N_4_/Bi_2_O_2_CO_3_**@**Pb^2+^ may have potential for energy-storage applications; therefore, its electrochemical properties should be investigated further. These assessments are necessary to support the safe, sustainable, and broader environmental application of CFA/g-C_3_N_4_/Bi_2_O_2_CO_3_ composite.

## Data Availability

The data will be made available on request.
